# Embryonic temperature has long-term effects on muscle circRNA expression and somatic growth in Nile tilapia

**DOI:** 10.3389/fcell.2024.1369758

**Published:** 2024-08-01

**Authors:** Golam Rbbani, Riaz Murshed, Prabhugouda Siriyappagouder, Fedor Sharko, Artem Nedoluzhko, Rajesh Joshi, Jorge Galindo-Villegas, Joost A. M. Raeymaekers, Jorge M. O. Fernandes

**Affiliations:** ^1^ Genomics Division, Faculty of Biosciences and Aquaculture, Nord University, Bodø, Norway; ^2^ Paleogenomics Laboratory, European University at Saint Petersburg, Saint Petersburg, Russia; ^3^ Paleogenomics Laboratory, National Research Center “Kurchatov Institute”, Moscow, Russia; ^4^ GenoMar Genetics AS, Oslo, Norway; ^5^ Institute of Marine Sciences, Spanish National Research Council, Barcelona, Spain

**Keywords:** developmental plasticity, thermal plasticity, non-coding RNAs, myogenesis, somatic growth, aquaculture

## Abstract

Embryonic temperature has a lasting impact on muscle phenotype in vertebrates, involving complex molecular mechanisms that encompass both protein-coding and non-coding genes. Circular RNAs (circRNAs) are a class of regulatory RNAs that play important roles in various biological processes, but the effect of variable thermal conditions on the circRNA transcriptome and its long-term impact on muscle growth plasticity remains largely unexplored. To fill this knowledge gap, we performed a transcriptomic analysis of circRNAs in fast muscle of Nile tilapia (*Oreochromis niloticus*) subjected to different embryonic temperatures (24°C, 28°C and 32°C) and then reared at a common temperature (28°C) for 4 months. Nile tilapia embryos exhibited faster development and subsequently higher long-term growth at 32°C compared to those reared at 28°C and 24°C. Next-generation sequencing data revealed a total of 5,141 unique circRNAs across all temperature groups, of which 1,604, 1,531, and 1,169 circRNAs were exclusively found in the 24°C, 28°C and 32°C groups, respectively. Among them, circNexn exhibited a 1.7-fold (log_2_) upregulation in the 24°C group and a 1.3-fold (log_2_) upregulation in the 32°C group when compared to the 28°C group. Conversely, circTTN and circTTN_b were downregulated in the 24°C groups compared to their 28°C and 32°C counterparts. Furthermore, these differentially expressed circRNAs were found to have multiple interactions with myomiRs, highlighting their potential as promising candidates for further investigation in the context of muscle growth plasticity. Taken together, our findings provide new insights into the molecular mechanisms that may underlie muscle growth plasticity in response to thermal variation in fish, with important implications in the context of climate change, fisheries and aquaculture.

## Introduction

Aquatic animals have been experiencing significant population declines and shifts in distribution over the last few decades, as a result of global climate change that challenges their tolerance limits (Barange et al., 2018; Chaudhary et al., 2021). Phenotypic plasticity is a property by which living organisms express different phenotypes in response to variable environmental conditions and it arises from complex interactions between genes and the environment (Burggren, 2019). Teleost, for example, exhibit an extraordinary capacity to dynamically adapt to a wide range of environmental changes, influencing various aspects of their biology, including swimming behavior, enzyme activity, metabolic rates, and muscle growth patterns (Macqueen et al., 2008; Burt et al., 2012; Schnurr et al., 2014).

Muscle growth plasticity is a crucial feature attributed to the ability of muscles to undergo indeterminate or continual growth throughout the lifespan. Teleost muscle growth involves both the recruitment of new muscle fibers (hyperplasia) and the enlargement of existing muscle fibers (hypertrophy). However, hypertrophy and hyperplasia are strongly influenced by genetic and epigenetic backgrounds and abiotic factors, such as temperature, oxygen level, photoperiod, diet, and microbiota (Johnston, 2006; Campos et al., 2013a; Chapagain et al., 2020; Skjærven et al., 2020).

Temperature significantly impacts growth as it varies due to short-term heat waves and long-term seasonal fluctuations in their aquatic environment (Alvarenga and França, 2009; Campos et al., 2013b; Dussenne et al., 2022). Teleosts have a narrow optimal temperature range during their developmental stages, where even minor temperature changes can significantly affect embryonic development and life history traits (Campos et al., 2014a, Rahman et al., 2021). The lasting effects of embryonic temperature on muscle development involve changes in the number of muscle precursor cells responsible for post-embryonic growth in teleosts (Green and Fisher, 2004; Kourkouta et al., 2021). For instance, zebrafish exposed to varying embryonic temperatures (22 °C, 26°C, and 31°C) exhibited distinct muscle fiber production patterns, with those at 26°C showing a 19% and 14% higher final fiber number compared to counterparts exposed to 22°C and 31°C, respectively (Johnston et al., 2009). In salmon, embryos initially raised at 10°C and then reduced to 5°C demonstrated accelerated early-stage growth in comparison to salmon consistently reared at 5°C (Burgerhout et al., 2017). However, the salmon raised at 5°C displayed sustained muscle growth over an extended period, accompanied by stimulated hyperplasia in the white muscle of larvae and juveniles.

Several studies have identified regulatory RNAs, such as long non-coding RNAs, miRNAs and piwi RNAs, as key mediators of the persistent effects of embryonic temperature on body growth and muscle cellularity. miRNAs play a vital role in acclimation and adaptation by modulating muscle-associated genes, and their expression is influenced by embryonic temperature (Blödorn et al., 2021; Raza et al., 2022). Among them, miR-1, miR-24, miR-17a, miR-133a, miR-181, miR-206, miR-499, and miR-214 are known to interact with the transcriptional networks involved in embryonic myogenesis in fish (Bizuayehu and Babiak, 2014). In Senegalese sole, higher incubation temperatures resulted in increased expression of several miRNAs (e.g., miR-17a, miR-181-5p, and miR-206-3p) that are positively correlated with growth at specific developmental stages (Campos et al., 2014b). Additionally, the temperature-sensitive miR-181c has been shown to regulate the transition between hyperplastic and hypertrophic muscle growth in zebrafish (*Danio rerio*) (Johnston et al., 2009).

Circular RNAs (circRNAs) are mostly non-coding RNA molecules produced via back-splicing, creating a covalent bond between the 3′- and 5′-ends of RNA transcripts. CircRNAs often show spatiotemporal characteristics and play pivotal roles in fine-tuning post-transcriptional gene expression through sequestering miRNAs and proteins (Jeck et al., 2013; Kulcheski et al., 2016). Thousands of circRNAs have been identified in the muscles of several terrestrial farmed animals, but the reports in fish remain relatively limited (Rbbani et al., 2021). Notable findings include the identification of 2,949 novel circRNAs in Nile tilapia and 445 in snout sea bream (*Megalobrama amblycephala*), highlighting their abundance and potential impact on fast muscle development (Liu et al., 2021; Rbbani et al., 2023). Additionally, Quan et al. (2021) have identified 324 differentially expressed circRNAs in rainbow trout (*Oncorhynchus mykiss*) subject to temperature stress. In particular, novel_circ_003889, novel_circ_002325, and novel_circ_002446 act in competing ceRNA networks (ceRNA) with miRNAs and mRNAs to enable adaptation to unfavorable conditions. It is plausible that circRNA expression may have a substantial long-term effect on thermal experience during early ontogeny, since recent studies have also shown that circRNA molecules can act in circRNA-miRNA-mRNA (ceRNA) networks to regulate various physiological and molecular processes (Rbbani et al., 2021; Yang et al., 2021; Rbbani et al., 2023). Despite this, the thermal developmental plasticity of the circRNA transcriptome and its connection to muscle growth remains unexplored. Nile tilapia (*Oreochromis niloticus*) is a popular aquaculture species worldwide due to its advantageous traits, such as rapid growth, acceptance of formulated feed, suitability for production in diverse systems and adaptability to a wide temperature range (El-Sayed and Fitzsimmons, 2023). This study aimed to investigate the long-term influence of embryonic temperature on circRNA expression in Nile tilapia, so as to gain insights into their potential involvement in developmental plasticity of growth in fish.

## Materials and methods

### Ethics approval

All animal handling procedures were performed in accordance with the EU Directive 2010/63 on the use of animals for scientific purposes, with approval from the Norwegian Animal Research Authority (FOTS ID 1042).

### Fish husbandry and artificial breeding

Fish were maintained in a recirculatory system with a water temperature of 28 °C, pH of 7.5, photoperiod of light:dark. 13 h:11 h, and dissolved oxygen of 8.33 mg/L (Podgorniak et al., 2019). During this period, they were fed commercial pellets (Skretting, Norway). For artificial breeding, one female was selected based on secondary sexual characteristics such as fin color, tender abdomen, and a prominent, hyperemic genital papilla, indicating their readiness for spawning. Subsequently, the fish was anesthetized by immersion in a clove oil (Sigma Aldrich, United States) emulsion containing 5 mL of a clove oil pre-mix in 96% ethanol (1:10 v/v) in 10 L of water. After measuring length and weight, the fish were given an intramuscular injection of human chorionic gonadotropin (hCG) at a dose of 2 international units (IU)/g. The injection was performed on the dorsal (upper) part of the fish above the lateral line and below the anterior part of the dorsal fin. hCG-injected females were kept in separate 50 L tanks with a water temperature of 28°C and a continuous air supply. After 24 h post-injection, one mature male and a hormone-injected female were anesthetized as above and dried around the vent with a soft towel to avoid residual anesthetic. Eggs were collected in a beaker by stripping through gentle rubbing of the abdomen. Soon after collecting the eggs, approximately 0.5 mL milt obtained by stripping was poured into the beaker. Fertilization was done through gentle shaking with the addition of 3 mL of recirculatory system water (28°C) into a 500 mL beaker for 8–10 min.

### Temperature experiment

**FIGURE 1:**
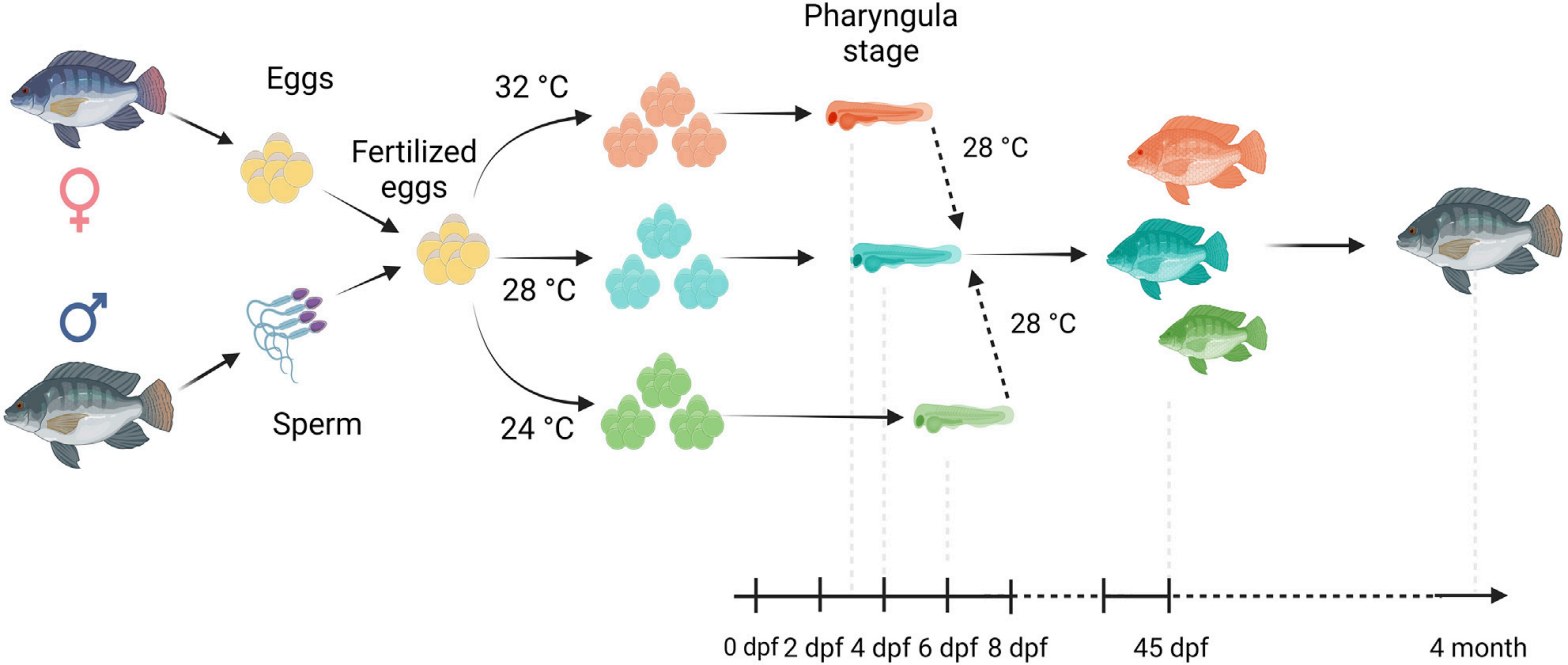
Overview of our experimental setup. Fertilized eggs were incubated at 24°C, 28°C or 32°C up to the opercular stage and subsequently transferred and reared at 28°C until the 4 month old. Here, days post fertilization is denoted as dpf.

Fertilized Nile tilapia eggs obtained as above were incubated at three temperatures (24 ± 0.5°C, 28 ± 0.5°C and 32 ± 0.5°C) in triplicate groups. These were selected to represent a low (24°C), medium (28°C) and high (32°C) embryonic temperature within the tolerance limit for normal development in Nile tilapia (Rana, 1990). Temperature in the tank was controlled with the heater (EHEIM precision aquarium heater; Germany) and monitored using EBI 300 temperature probe (Ingolstadt, Germany). The eggs were washed and distributed randomly in three different tanks (500 L) containing three egg rockers (5 L) each. Each egg rocker contained 300 embryos. A continuous flow of system water into the egg rockers was maintained through a small pump, and the initial temperature of the tanks was maintained at 28 ± 0.5°C. One tank had its temperature increased to 32°C, while the other had its temperature decreased to 24°C with a gradient of ∼0.5°C every hour (Supplementary Figure S1). Water salinity, O_2_, pH, and nitrogenous compounds were periodically monitored throughout the entire trial and larvae were exposed to an artificial photoperiod of 13 h:11 h (light:dark). Rearing tanks were continuously monitored to remove unfertilized and dead eggs. In order to monitor the developing embryos, they were precisely mounted on a Petri dish and secured in place using 3% methylcellulose (Sigma). Lower magnification images, capturing the complete specimens, were then captured using a Leica MZ16 FA dissecting stereomicroscope (Leica Microsystems GmbH, Wetzlar, Germany). When the embryos completed the pharyngula stage (Fujimura and Okada, 2007), they were transferred to 28°C, fed *ad libitum* with Amber Neptun pellets (Skretting, Norway) from the mouth opening stage. Fish from all embryonic temperature groups were reared until the adult stage at 28°C, since the optimal temperature for Nile tilapia growth ranges from 27°C to 30°C (Nivelle et al., 2019) and reared until they reached the adult stage. The development and growth of the embryos and larvae were monitored throughout the experiment (Figure 1).

### Sample collection

At the juvenile stage (45 dpf), the weight of all fish from each temperature group was measured, i.e. 244, 250 and 232 from 24°C, 28°C and 32°C, respectively. The morphometric data of the nine individuals from each group that were randomly selected for transcriptomic analysis are indicated in Supplementary Table S6. The fish were euthanized using a 10 mL solution of clove oil (Sigma Aldrich, United States) mixed in a 1:10 ratio with 95% ethanol, which was then diluted in 10 L of water. Subsequently, fast muscle was dissected from a cross-section taken at 0.7 standard length, just anterior to the anus (Supplementary Figure S2), as described in Fernandes et al. (2005). Muscle samples were snap-frozen in liquid nitrogen and stored at −80°C until RNA extraction.

Fast muscle steaks from randomly selected fish were cut into blocks measuring 0.8 cm side length, embedded in Cryomatrix™ resin (Thermo Fisher Scientific, United States) and frozen for 60 s in 2-methylbutane (Sigma-Aldrich, United States) cooled in a liquid nitrogen bath. The frozen muscle blocks were then stored in liquid nitrogen at −80°C until histological analysis.

### RNA extraction

Total RNA extraction from fast muscle samples (45 dpf) was performed using the Direct-zol™ RNA MiniPrep kit (Zymoresearch, CA, United States), following the manufacturer’s instructions. To determine the concentration of RNA in the samples, a Nanodrop spectrophotometer (Nanodrop Technologies/Saven Werner) and Invitrogen Qubit 3.0 fluorometer (ThermoFisher Scientific, United States) were used. The quality of the RNA was evaluated using the TapeStation 2,200 (Agilent Technologies, United States), and only samples with RNA integrity number (RIN) value greater than seven were kept for the subsequent library preparation.

### Depletion of linear RNA and rRNA

One microgram of total RNA was used as starting material for circRNA enrichment. Linear RNAs were removed with the highly efficient 3′–5′ exonuclease ribonuclease R (LCG Biosearch Technologies, Germany). Specifically, 2 units of RNase R were used in a 20 μL reaction containing 2 μL of 10× RNase R buffer at 37°C for 30 min. The linear RNA-free residue was cleaned up with RNA cleaning beads following the manufacturer’s protocol. The clean RNA was treated with the NEBNext^®^ rRNA Depletion Kit v2 (NEB, United States) to remove ribosomal RNA (5S, 5.8S, 18S, and 28S) according to the manufacturer’s protocol. The procedure involved using a hybridization probe to bind unwanted ribosomal RNAs, followed by RNase H digestion to degrade the hybridized RNA by recognizing RNA: DNA bonds. Finally, DNase I was used to digest the DNA probes, and the remaining reaction was cleaned using magnetic beads (NEB, United States).

### Library preparation and sequencing

CircRNA libraries are prepared using the NEBNext^®^ Ultra™ II Directional RNA Library Prep Kit using the manufacturer’s protocol (United States). In brief, circRNA-enriched RNAs (RNAse R+ rRNA-) were fragmented into short sequences (>200 nt) in buffer at 94 °C for 12 min using first-strand reaction buffer and random primer mix (NEB, United States). The first-strand cDNA was transcribed from fragmented RNA using random hexamers. Subsequently, the second-strand cDNA was synthesized, and cDNA fragments were purified using the AMPure XP beads (Beckman Coulter, Brea, CA, United States). Then, any damaged ends of the double-stranded cDNAs were repaired, and the cDNA fragments were prepared for adapter ligation. After ligating the sequencing adapters, size selection was performed using magnetic beads to ensure that only properly sized fragments were retained. Finally, PCR amplification (16 cycles) was carried out using Phusion high-fidelity DNA polymerase, a universal PCR primer, and barcoded index primers to amplify the libraries. The libraries were purified once more using AMPure beads, and their quality was assessed on the TapeStation 2,200 (Agilent, United States) using high sensitivity D1000 tape. All 27 libraries, representing different temperature groups (n = 9 for 24°C, 28°C, and 32°C), were pooled together. The pooled library was loaded onto the Illumina NovaSeq 6,000 Sequencing system (Illumina, San Diego, CA, United States) to generate 100 base pair paired-end read. The sequencing was conducted at the high-throughput sequencing facility at the Novogene sequencing center in the United Kingdom.

### *In silico* analysis of circRNAs

The quality of raw reads (both Q20 and Q30) was assessed using fastqc (v0.11.5), and subsequently, fastp (v0.19.10) was employed to filter out adapters, poly-N, unknown nucleotides, and trimmed low-quality reads (bases with a quality value Q < 20) (Chen et al., 2018). The resulting high-quality, clean sequences were used for downstream analysis. The Nile tilapia reference genome (O_niloticus_UMD_NMBU assembly GCA_001858045.3) was obtained from the National Center for Biotechnology Information (NCBI) and CIRIquant was employed for circRNA identification and quantification. CIRIquant utilizes BWA, HISAT2, and StringTie index of the reference sequence for read alignment (Zhang et al., 2020). CIRIquant filters out reads with strong evidence of spliced alignment (minimum mapped segment length >5 bp) and uses unmapped reads as candidates for circRNA detection. By default, CIRIquant uses CIRI2 for circRNA detection. To achieve accurate quantification of circRNAs, CIRIquant generates a pseudo-reference of the circular sequence by repeating the full length of the back-spliced region twice and read pairs that map concordantly across a 10 bp region from the junction site are identified as circular RNAs. To determine common and embryonic temperature-specific circRNAs, we combined all circRNAs from each temperature group, compared the lists and represented the results as a Venn diagram. CircRNAs that were found exclusively in one group (not necessarily in all biological replicates) but absent in others were considered specific to that embryonic temperature group.

### Functional annotation of circRNA host genes

To better understand the potential biological functions of the identified circRNA-producing genes, we performed a comprehensive functional annotation using Gene Ontology (GO) terms and Kyoto Encyclopedia of Genes and Genomes (KEGG) pathways. The list of host genes producing developmental temperature-specific circRNAs was imported into clueGO- a Cytoscape (v 3.8.2) plug-in. Then, we annotated the genes on GO and KEGG databases. To reduce the false positive rate, an adjusted *p*-value ≤0.05 (Benjamini–Hochberg multiple testing) was used as a threshold significance level for functional enrichment. Additionally, we performed functional gene set analysis (GSA) and organized the enriched functional categories into functionally grouped networks, highlighting the biological relationships between them (Shannon et al., 2003).

### Differential expression analysis of circRNAs

To perform differential expression analysis of circRNAs, we used back-splice junction reads from identified circRNAs as expression count and created a matrix using circMeta (Chen et al., 2020). The expression levels were estimated using counts per million mapped reads (CPM) and normalized by sequencing depth and length. Next, we applied the Poisson-based test (z-test) from circMeta to identify differentially expressed circRNAs among different temperature groups. The *p*-values obtained were corrected using the Benjamini–Hochberg method for multiple testing. CircRNAs displaying p-adjusted value < 0.05 and |log_2_fold-change| ≥1 were considered as differentially expressed.

### miRNA target prediction

Given the ability of circRNAs to act as miRNA sponges, we conducted miRNA target prediction for the differentially expressed circRNAs. We obtained mature miRNA sequences of Nile tilapia from miRbase v22.1 (Kozomara et al., 2019) and used the miRanda (v3.3a) software, which is a widely accepted miRNA target prediction tool to identify circRNA and miRNA interactions (Enright et al., 2003). CircRNA-miRNA pairs with a pairing score >120 and an energy score < −20 kcal/mol were considered true targets.

### CircRNA-miRNA network construction

The Cytoscape software (v3.8.2) was used to visualize circRNA-miRNA networks (Shannon et al., 2003). Our approach involved importing predicted miRNA targets and differentially expressed circRNAs into Cytoscape and the use of the “miRNA-target” plugin for mapping circRNAs to their respective miRNA targets. To organize the nodes in a circular layout, we employed the yFiles Layout Algorithms plugin. Finally, we manually highlighted the most significant nodes and connections involved in growth-related processes.

### Muscle histology

Fast muscle samples (n = 9 per group) from three temperature groups were processed for histological analysis. Seven micra-thick sections were prepared from cytomatrix blocks using a microtome (Microm HM355S, Germany) at −20 °C. After air-drying, the tissue sections were stained with hematoxylin and eosin using standard procedures (Campos et al., 2013a). Microscopic photomicrographs were obtained using the Leica DM3000 LED microscope (Leica Camera AG, Wetzlar, Germany) equipped with a Leica MC 190HD camera. Muscle fiber analysis was conducted on approximately 1,000–1,100 fibers from both the dorsal (epaxial) side of the white muscle slice in each fish using Axio Vision software (Rel.4.2, Carl Zeiss INC., Germany), with a focus on measuring muscle fiber diameters (μm).

### RT-qPCR validation

Primers and PCR conditions for circRNA validation were obtained using the circPrime web tool (Sharko et al., 2023). Complementary DNA (cDNA) was synthesized from 1,000 ng of total RNA from the same samples used for RNA library preparation, using the QuantiTect Reverse Transcription Kit (Qiagen, Germany). The resulting cDNA was further diluted 10-fold with nuclease-free water and used as the PCR template. Divergent primers were designed to amplify the circRNA fragment across the junction point (Table 1).

**TABLE 1:** List of primer sequences of differentially expressed circRNAs.

circRNA	Primer sequence (5′–3′)	Annealing temperature(°C)	Amplicon size (bp)
circNexn	Forward: TGC​GCT​CTT​CCT​CTA​CCC​T	60	180
	Reverse: AGA​GGC​TGT​TTG​CTG​AGG​C		
circTTN	Forward: AGG​AGA​CGG​TGG​AGA​CCA​A	60	180
	Reverse: GGG​GAA​AGC​AGA​GGA​TGG​G		
circTTN_b	Forward: AAA​CTG​CCA​GCT​GGA​CCA​G	60	180
	Reverse: CCT​CCT​GGT​GCT​TGG​ACT​G		

A Bio-Rad CFX96 real-time thermocycler (Bio-Rad Laboratories, Hercules, CA, United States) was used for qPCR amplification under the following conditions: an initial enzyme activation/cDNA denaturation step at 95°C for 1 min, followed by 45 cycles at 95°C for 15 s, 60°C annealing for 15 s, and 72°C for 15 s, with a final standard dissociation protocol to obtain the melting profiles. The relative expression levels of the target circRNAs were calculated using the ΔΔCT method, with *actin beta* and *elongation factor 1-alpha* as reference genes.

## Results

### Developmental temperature has a long-term effect on growth and muscle cellularity in nile tilapia

**FIGURE 2:**
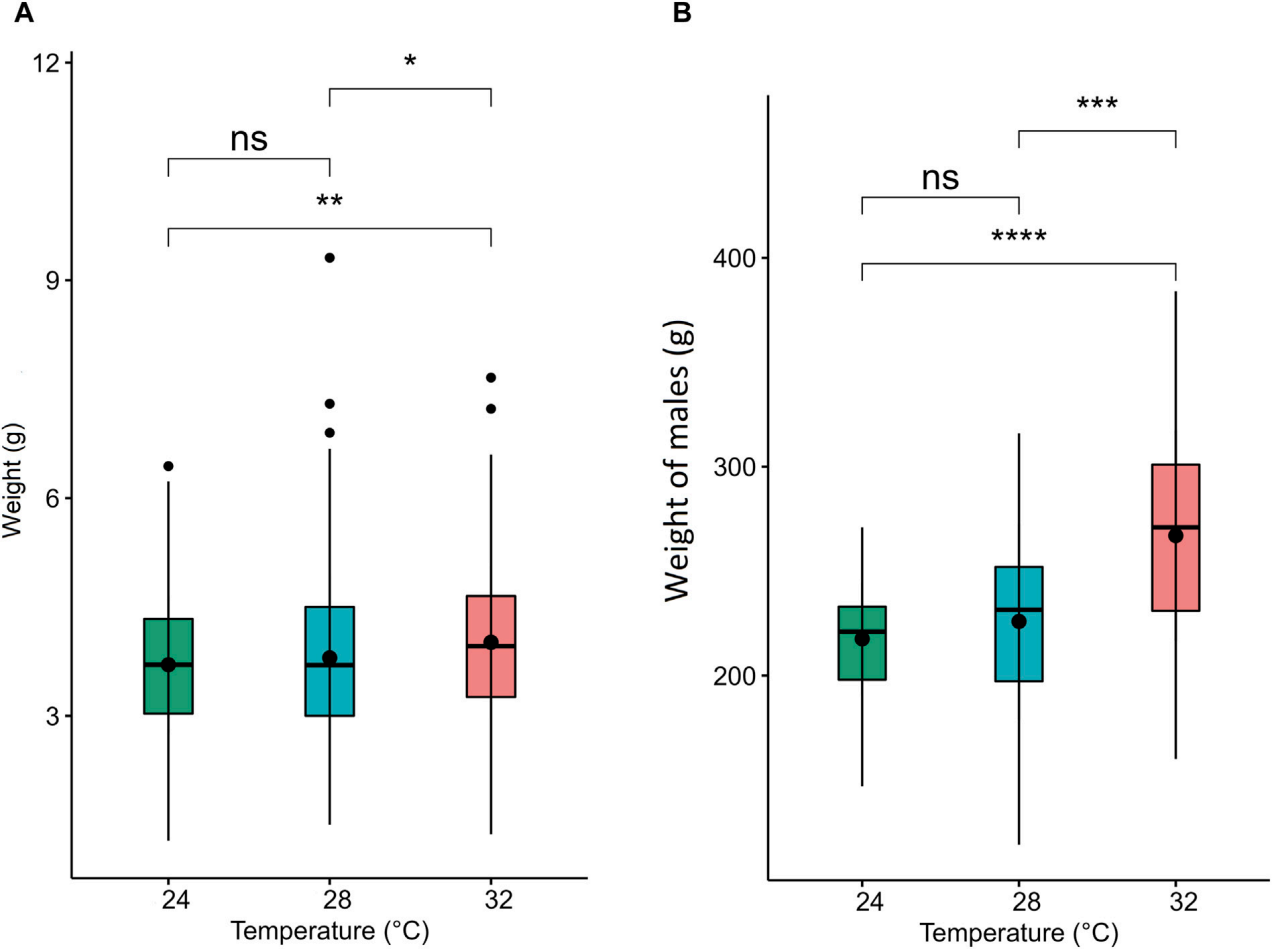
Weight and muscle fiber distribution in Nile tilapia reared at different embryonic temperatures. The boxplots show the weight of the fish at **(A)** 45 dpf and **(B)** 4.5 months post-fertilization. 24°C, 28°C and 32°C groups are denoted in green, blue, and red, respectively. Black dots inside each box indicate the mean weight values. ****,***,**, * and ns indicate *p* < 0.0001, *p* < 0.001, *p* < 0.01, *p* < 0.05 and non-significant, respectively.

The water quality parameters were considered appropriate for Nile tilapia culture. Supplementary Table S1 represents water parameters during the experimental period. Nile tilapia embryos displayed temperature-dependent development, characterized by accelerated growth at higher water temperatures. Embryos incubated at 32°C, 28°C, and 24°C reached the pharyngula stage at 70, 90, and 144 h post-fertilization (hpf), respectively (Table 2). However, temperature did not impact the survival of newly hatched larvae (Supplementary Table S2). Importantly, developmental temperature significantly influenced the long-term growth performance of Nile tilapia juveniles and adults. Statistical analysis (Wilcoxon test) revealed a substantial effect of developmental temperature on fish growth after 45 days post-fertilization (dpf), with no observable sexual difference at this stage (Figure 2A). Notably, a 4°C difference in temperature resulted in 5.57% and 8.31% higher mean weights for fish reared at 32°C compared to their counterparts at 28°C and 24°C, respectively. However, no significant difference was observed between the 28°C and 24°C groups. Moreover, this growth disparity persisted after sexual differentiation (4 months post-fertilization) (Figure 2B), with males exhibiting an even more prominent growth trend, achieving 18.2% and 22.8% higher mean weights at 32°C compared to the 28°C and 24°C groups. Moreover, our comprehensive linear model analysis underscored the pivotal role of weight at 45 dpf as a significant factor contributing to the observed differences in mean weight at 4 months (Supplementary Table S3).

**TABLE 2:** Developmental stages of Nile tilapia at different developmental temperatures. Development stages are defined as in (Fujimura and Okada, 2007). Scale bar indicates 1 mm.

Developmental stage		Hours post-fertilization (hpf)		
		24°C	28°C	32°C
Cleavage	2-cell 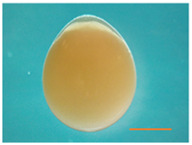	1.5	1.5	1.5
	8-cell 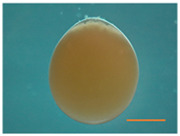	3.5–5	3.4	3.4
Blastula	Early blastula 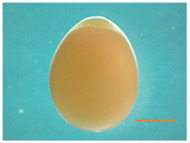	25	16	14
Gastrula	Epiboly (30%–90%) 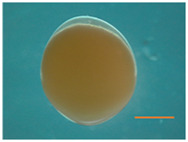	37	28	18
Segmentation	Yolk plug closure and somitogenesis 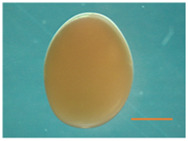	48	33	25
	Brain differentiation 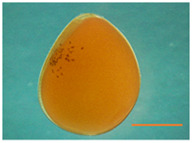	74	42	31
Pharyngula	Onset of blood circulation 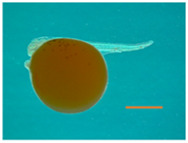	110	68	45
	Head enlargement 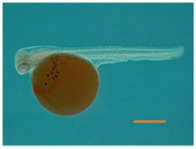	144	96	70
Hatching	Hatching 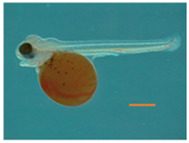	200	128	96

**FIGURE 3:**
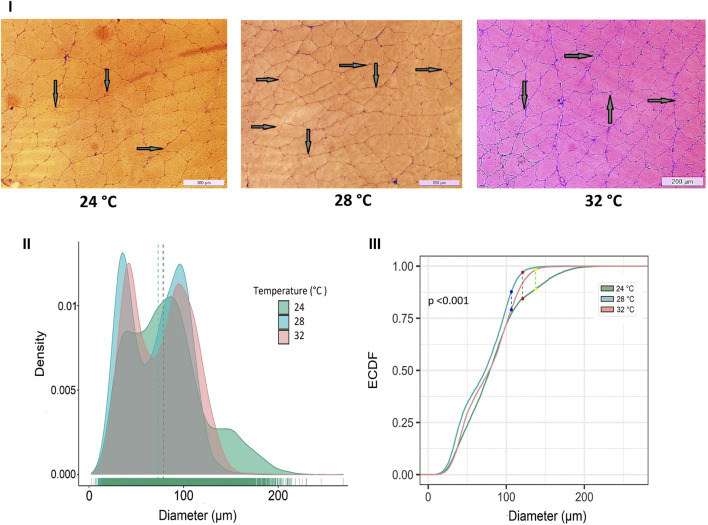
Differences in micromorphology in fast muscle of Nile tilapia treated at different developmental temperature. i) Representative histological images of muscle sections from 24°C, 28°C, and 32°C groups (Scale bar = 200 µm). The arrows in the image indicate newly recruited muscle fibers. ii) Probability density distribution of white muscle fiber diameters in 45 dpf Nile tilapia. The dotted lines represent the median of each group. (iii) The empirical cumulative distribution function (ECDF) curves of the Kolmogorov-Smirnov test along with maximum distance between distributions of muscle fiber diameters from 24°C, 28°C and 32°C.

Developmental temperature at embryonic stages also had a significant impact on fast muscle cellularity, particularly in fiber diameter and size (Figure 3). The muscle fibers of Nile tilapia subjected to three developmental temperatures displayed distinct differences in appearance when observed in cryomatrix-mounted sections stained with hematoxylin and eosin (Figure 3i). At 45 days post-fertilization (dpf), the probability density distribution of muscle fiber diameter indicated heterogeneity in fiber size. Mainly, the 24°C group exhibited a significant portion of large fibers, leading to a right-hand tail in the distribution, while the 28°C group had more on the left-hand tail (Figure 3ii). These findings were further supported by a Komolgorov-Smirnov statistical test, demonstrating significant differences (*p* < 0.001) between the fiber distributions of different temperature groups (Figure 3iii).

### Identification and characterization of circRNAs in fast muscle of nile tilapia exposed to different temperatures during embryogenesis

**FIGURE 4:**
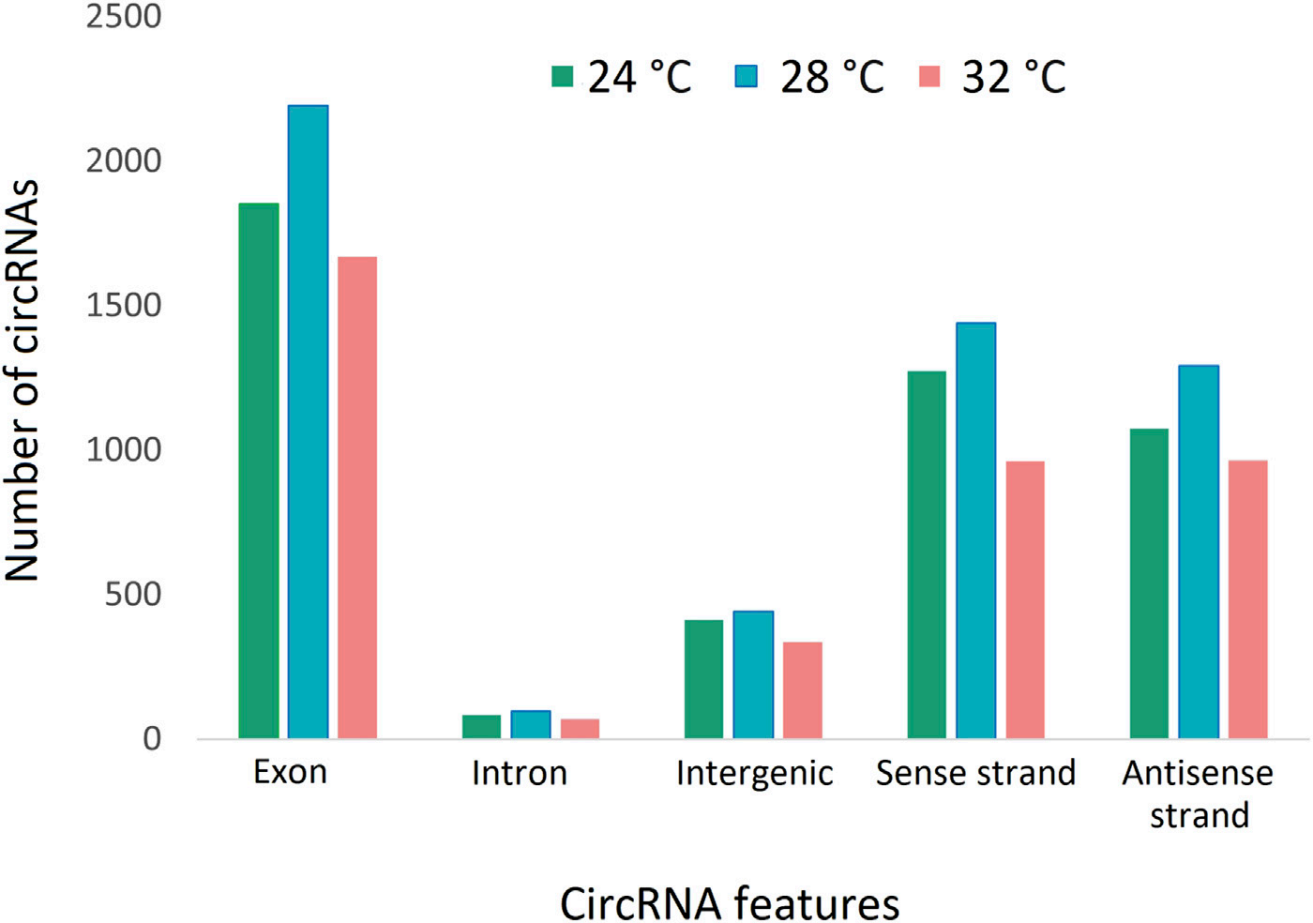
CircRNAs and their origins in three temperature groups of juvenile Nile tilapia. Each bar represents the number of circRNAs transcribed from exons, introns, intergenic regions, sense strand and antisense strand.

**FIGURE 5:**
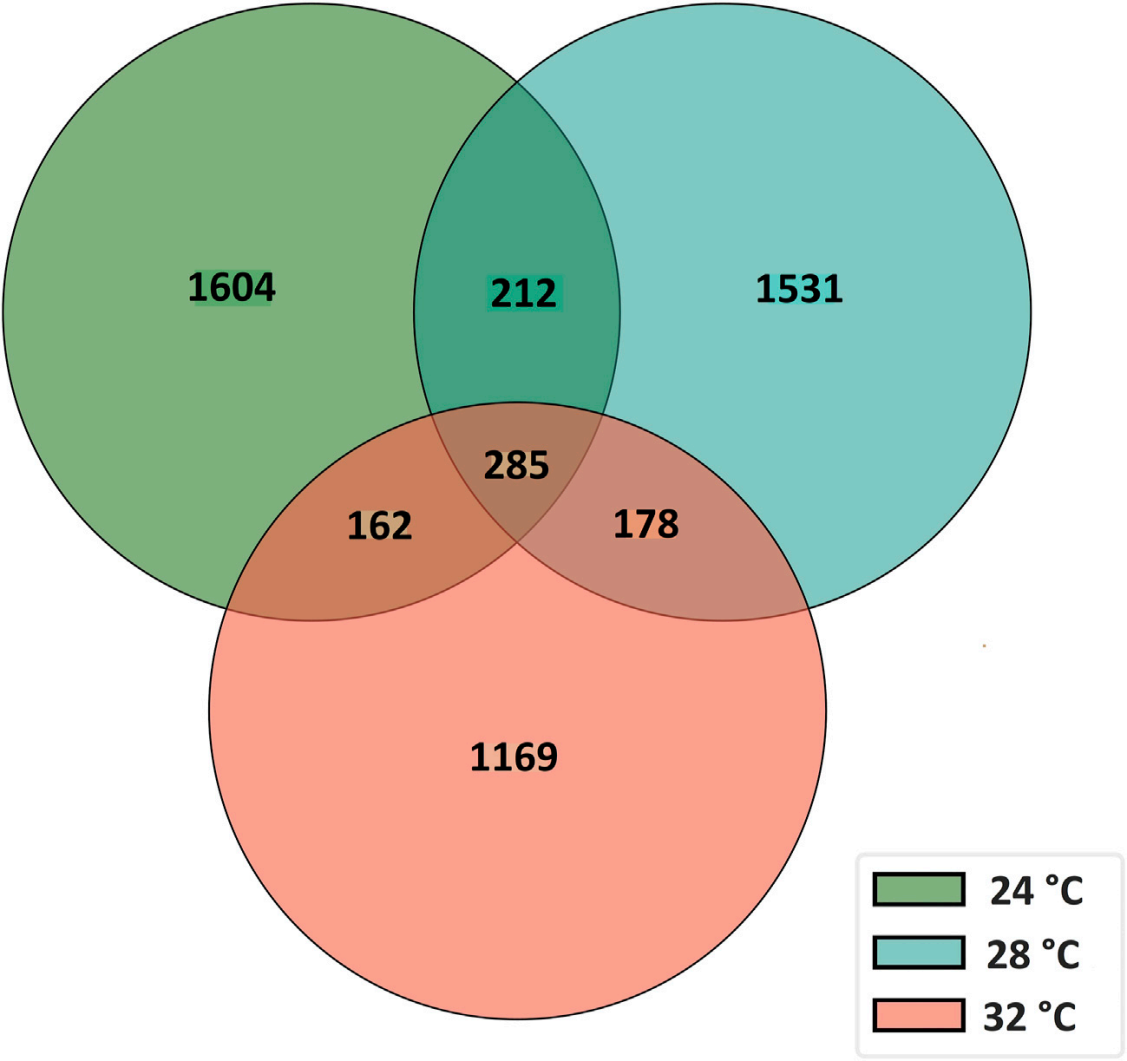
A Venn diagram showing the number of common and unique circRNAs in fast muscle of juvenile Nile tilapia whose embryos were incubated at 24°C, 28°C or 32°C until the completion of pharyngula stage.

To characterize the overall circRNAome, we profiled circRNAs in the fast muscle of Nile tilapia (45 days old) from all temperature groups. A total of 4,008.1 million raw sequencing reads were obtained from 24 samples from the three temperature groups. After pre-processing, 3,880.8 million reads (quality > Q20) were used for circRNA detection (Supplementary Table S4). Using CIRIquant, we identified between 159 and 562 distinct circRNAs in each library (Supplementary Figure S3). It is unusual that most circRNAs harbored non-GT/AG signals in Nile tilapia, and the most prominent splicing signals were TG/AG, TT/AG and TC/AG (Supplementary Figure S4). The analysis of circRNA distribution across the genome revealed that the highest number of circRNAs are produced from LG16 and LG7 chromosomes (Supplementary Figure S5). The majority of circRNAs were produced from exons (80%), followed by intergenic regions (16.6%) and introns (7.5%) (Figure 4). Surprisingly, we discovered a high proportion of circRNAs encoded by the antisense strand (46.5%). Further analysis revealed that each host gene produced, on average, between one and seven circRNAs. We then investigated how circRNA expression was influenced by temperature during early development. Overall, fish from the low-temperature group expressed the maximum number of circRNAs in total, whereas the high-temperature group had the lowest. A total of 285 circRNAs were common to all temperature groups, whereas 1,604, 1,531, and 1,169 circRNA were only expressed in 24°C, 28°C, and 32°C groups, respectively (Figure 5).

### The functions of the host gene of developmental temperature-specific circRNAs are associated with muscle growth plasticity

**FIGURE 6:**
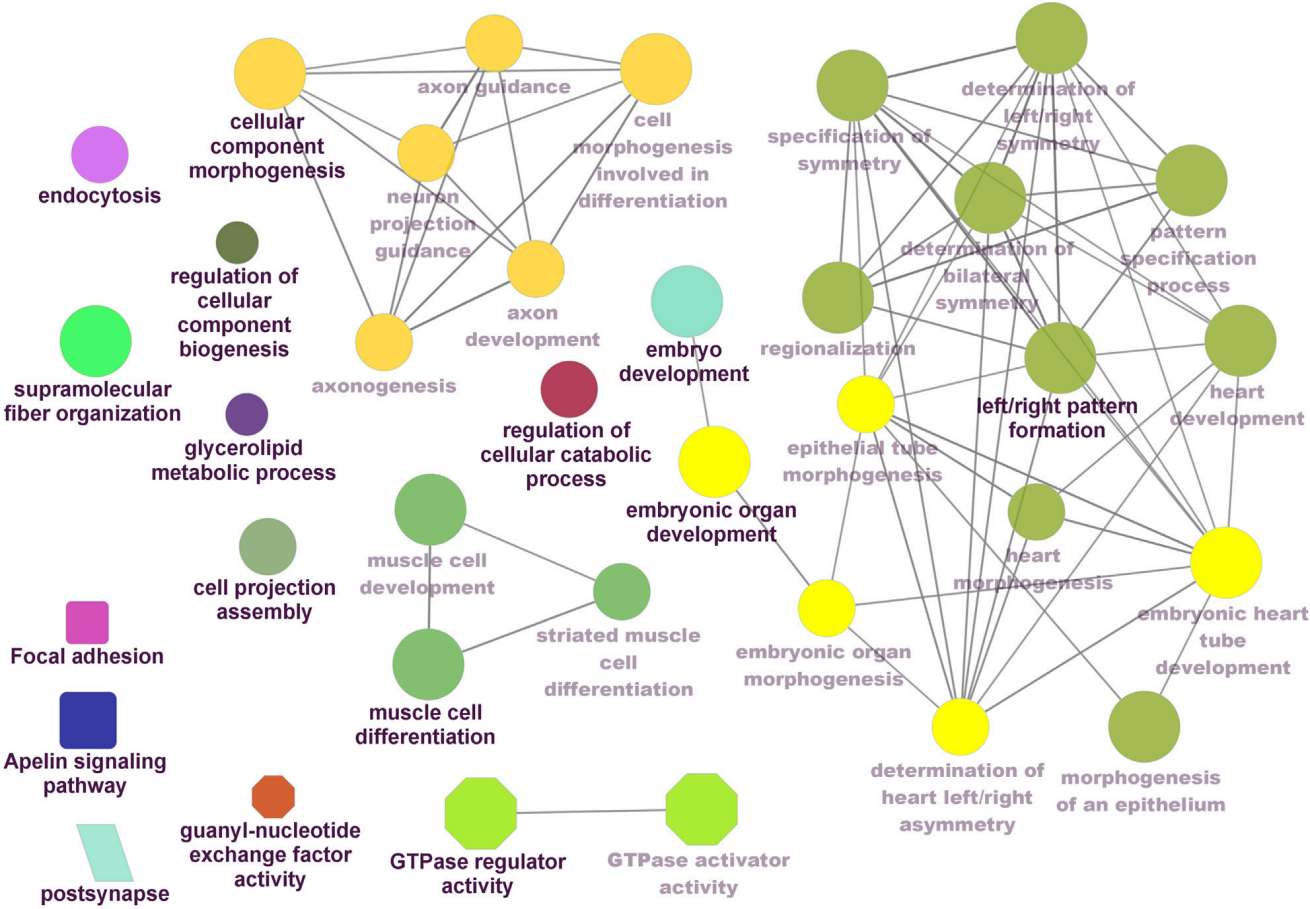
ClueGO enrichment analysis of overrepresented circRNA host genes from the 24°C group, focusing on Gene Ontology (GO) and Kyoto Encyclopedia of Genes and Genomes (KEGG) terms. Rectanglular nodes represent KEGG pathways, ellipses indicate biological processes, octagons correspond to molecular functions, and parallelograms represent cellular components.

**FIGURE 7:**
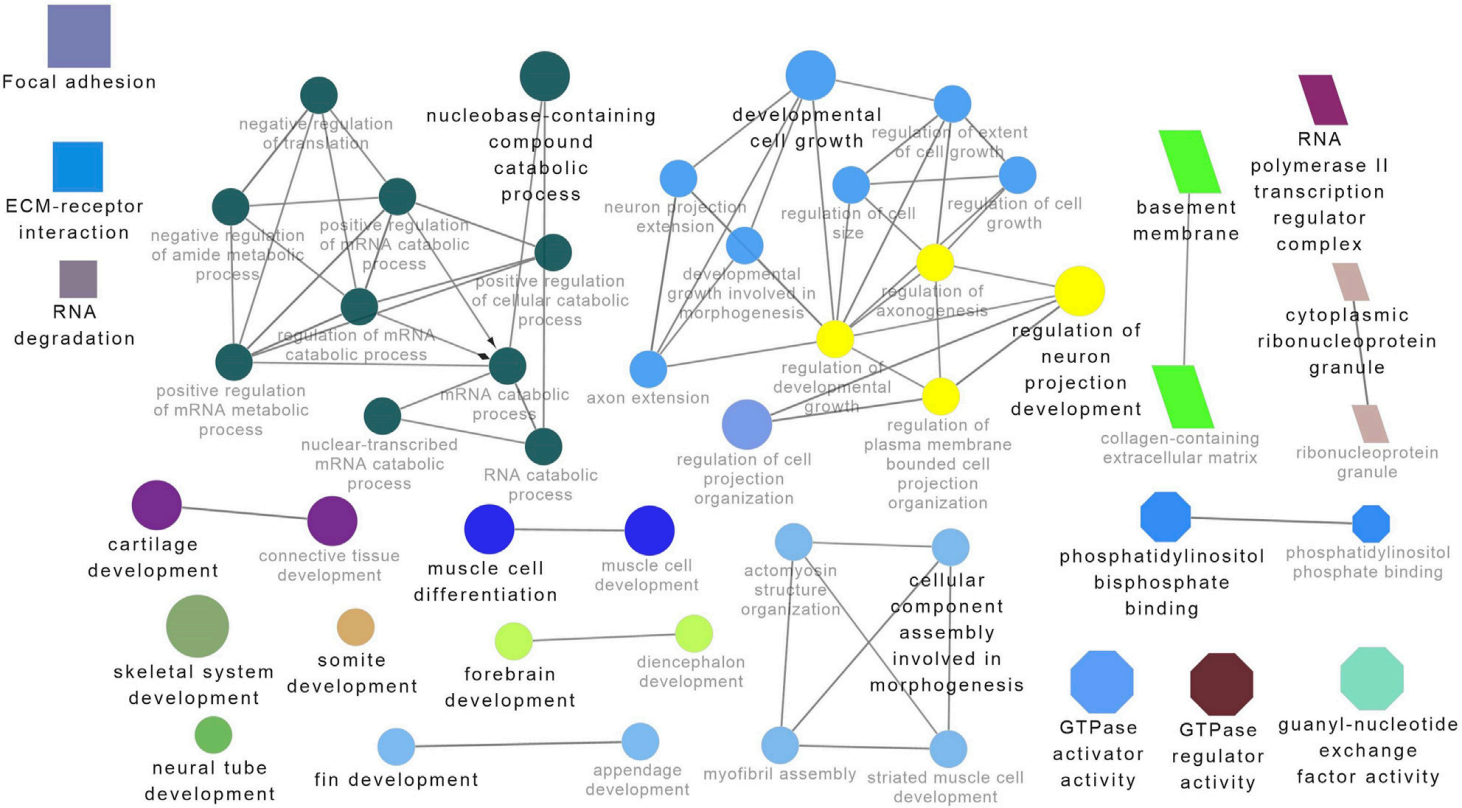
ClueGO enrichment analysis of overrepresented circRNA host genes from the 28°C group, focusing on Gene Ontology (GO) and Kyoto Encyclopedia of Genes and Genomes (KEGG) terms. Rectangle nodes represent KEGG pathways, ellipses represent biological processes, Octagons represent molecular functions, and parallelograms represent cellular components.

**FIGURE 8:**
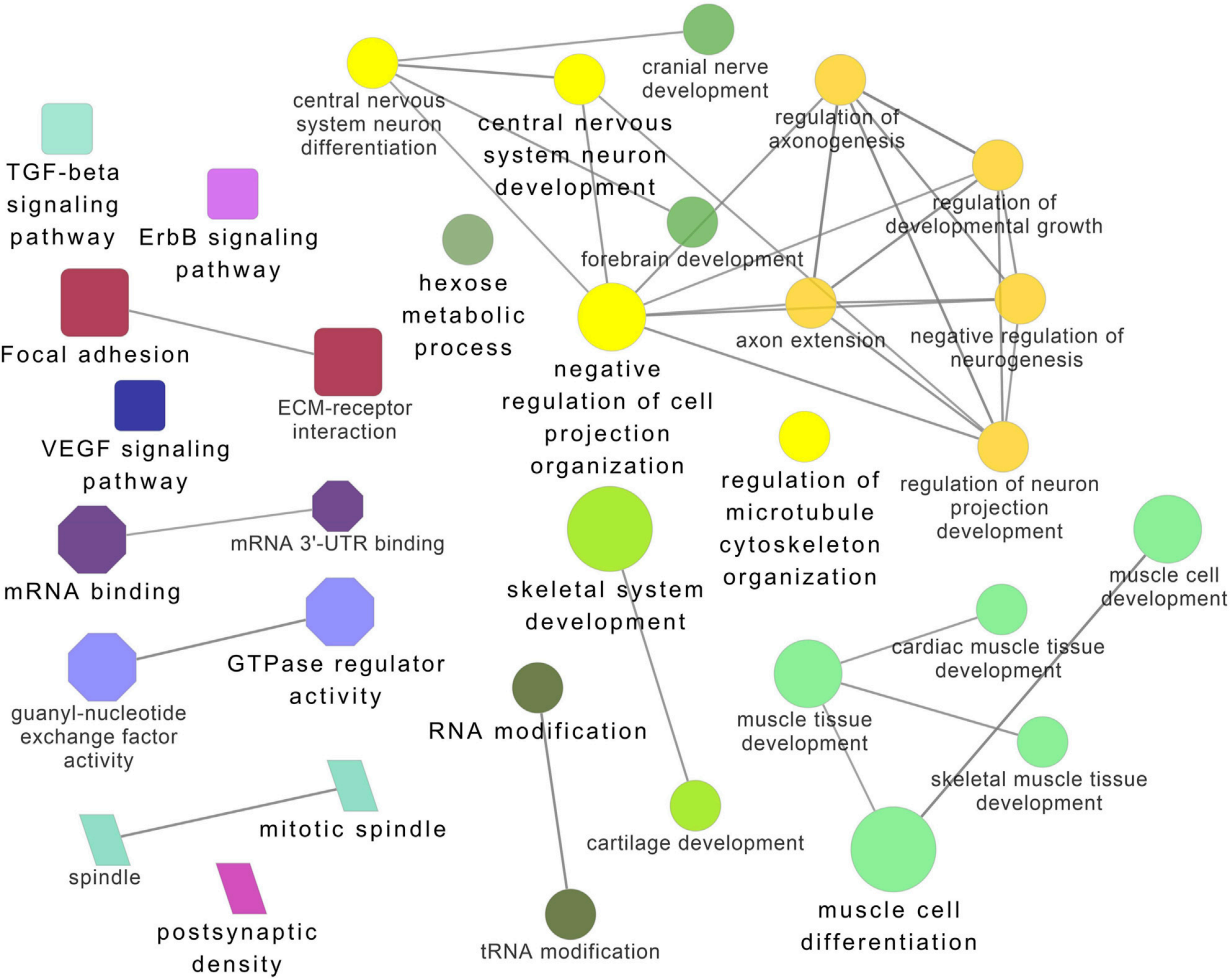
ClueGO enrichment analysis of overrepresented circRNA host genes from the 32°C group, focusing on Gene Ontology (GO) and Kyoto Encyclopedia of Genes and Genomes (KEGG) terms. Rectangle nodes represent KEGG pathways, ellipses represent biological processes, Octagons represent molecular functions, and parallelograms represent cellular components.

Functional enrichment analysis using Gene Ontology (GO) for the host genes of temperature-specific circRNAs revealed that the most significantly enriched (*p-adjusted < 0.05*) terms and pathways corresponded to developmental and metabolic processes, cell differentiation, muscle structure development, muscle cell development, protein binding, transcription factor binding, ubiquitin-binding, transcription coregulator activity, cyclin A2-CDK2 complex, catalytic complex, transferase complex (Figures 6–8). Metabolic pathway analysis by KEGG revealed that focal adhesion, apelin signaling, and ECM-receptor interaction pathways were related to growth and development (Figures 6–8). These findings suggest that these differentially expressed circRNAs may play an important role in muscle growth plasticity.

### CircRNAs derived from genes involved in muscle growth are strongly influenced by developmental temperature

To explore the potential role of circRNAs in temperature-mediated responses in Nile tilapia muscle, we conducted a comparative analysis of circRNA expression levels at three different temperatures: 24°C, 28°C, and 32°C (Supplementary Figure S6; Supplementary Table S7). The list of differentially expressed circRNAs (DE-circRNAs) and their details can be found in Table 3. Notably, circRNA NC_031986.2:45119249|45119970 (circNexn) exhibited a 1.7-log_2_fold up-regulation (p adjusted <0.05) at 24°C and a 1.3-log_2_fold up-regulation (p adjusted <0.05) at 32°C when compared to the 28°C group (Supplementary Figures S6A, S6B). CircNexn is an exonic circRNA originating from the *nexilin* gene. *The nexilin* gene encodes a protein that binds to *F-actin* and is involved in cell motility. Additionally, circRNA NC_031987.2:33760274|33761533 (circTTN) displayed a 1.1-log_2_-fold downregulation (p adjusted <0.05) in muscle of fish from the 24°C embryonic temperature group compared to the 28°C group (Supplementary Figure S6A). CircTTN is also an exonic circRNA derived from the *titin* gene, one of the longest genes in vertebrates and known for regulating thin and thick filament formation in muscle. Moreover, NC_031987.2:33769019|33776526 (circTTN_b), another circRNA derived from *titin* but at a different location, showed a 1.1-log_2_-fold downregulation in the 24°C group compared to the 32 °C group (Supplementary Figure S6C). These differentially expressed circRNAs are likely to have significant regulatory roles in developmental temperature-mediated responses in Nile tilapia muscle.

**TABLE 3:** Differentially expressed circRNAs in fast muscle of Nile tilapia whose embryos were incubated at 24 °C, 28 °C or 32 °C until completion of pharyngula stage.

Expression comparison (°C)	CircRNA NCBI ID	CircRNA name	CircRNA host gene	log_2_ fold change	adjusted *p*-value
24 °C vs. 28	NC_031986.2:45119249|45119970	CircNexn	*nexn*	1.70	9 E−05
	NC_031987.2:33760274|33761533	CircTTN	*LOC100702396(titin)*	−1.13	0.017
32 °C vs. 28	NC_031986.2:45119249|45119970	CircNexn	*nexn*	1.33	0.007
24 °C vs. 32	NC_031987.2:33769019|33776526	CircTTN_b	*LOC100702396* (*titin*)	−1.07	0.017

### Validation of differentially expressed circRNAs

All three differentially expressed circRNAs were quantified using RT-qPCR using divergent primers (approximately 20 nucleotides) that exclusively amplify the circRNA junction region (Table 3). The relative expression patterns of three circRNAs were consistent with the trends observed in the circRNA sequencing data (Supplementary Figure S7).

### Exploring potential muscle-related miRNA targets in differentially expressed circRNAs and their complex circRNA-miRNA networks

One of the primary focuses of circRNA functional studies is their role as miRNA sponges. Using the miRanda software, we identified a total of 314 targeted miRNAs with 493 possible interaction sites within the three differentially expressed circRNAs (Supplementary Table S5). Multiple muscle-associated miRNAs, also known as myomiRs, including miR-1, miR-9a, miR-24a, miR-34, miR-101, miR-133a, miR-143, miR-144, miR-181a, miR-190b, miR-202, miR-206, and miR-449, were predicted to interact with the differentially expressed circRNAs. Furthermore, we observed that circRNAs produced from titin (circTTN and circTTN_b) have multiple binding sites for the same miRNA and are able to bind to several myomiRs.

**FIGURE 9:**
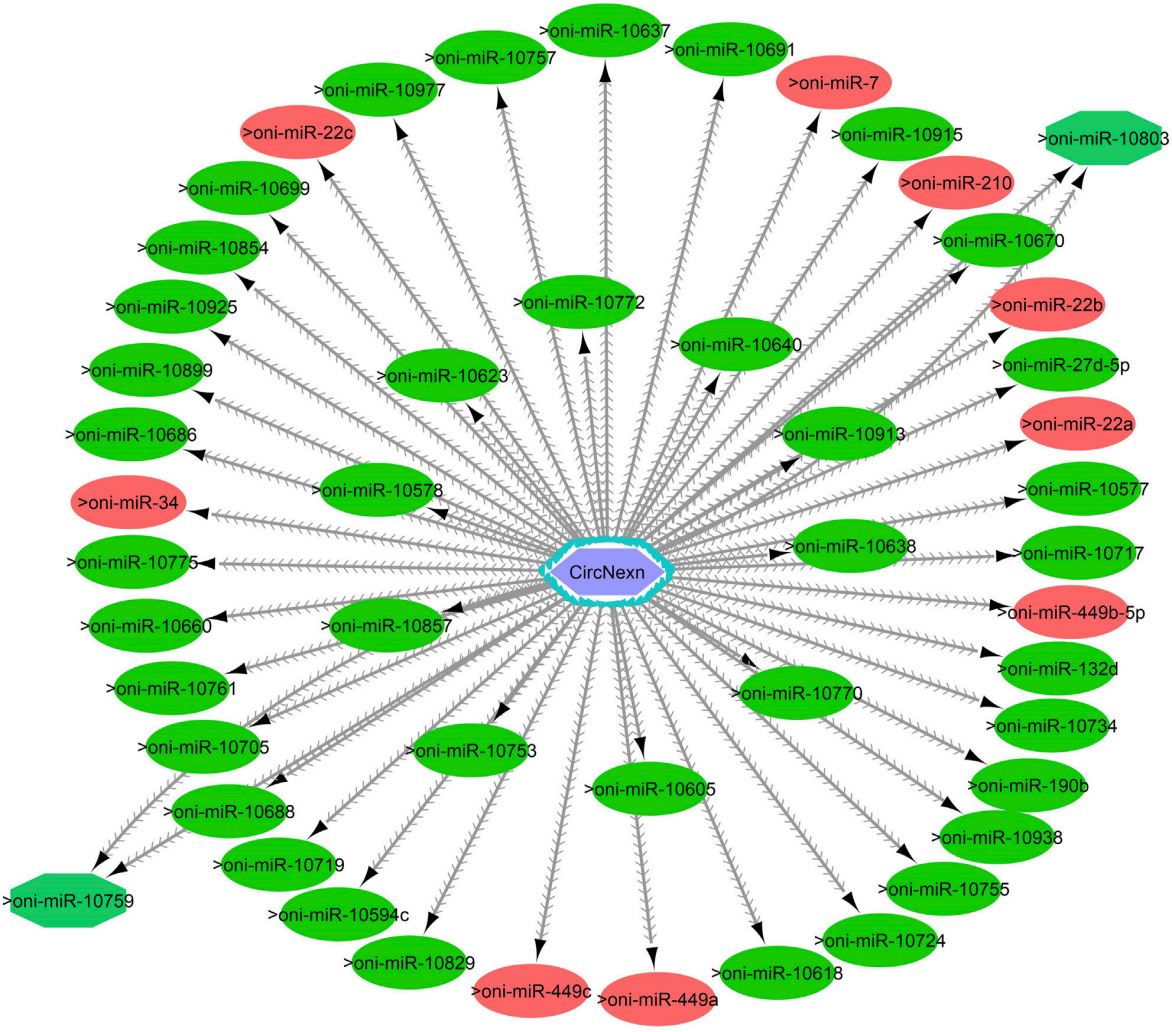
CircNexn-miRNA interaction network regulating the plasticity of muscle growth. Light blue hexagonal nodes represent circRNAs, and circles (single interaction) and octagonal nodes (multiple interactions) represent miRNAs. The potential circRNA-miRNA interactions that could be involved in thermal plasticity of muscle growth are highlighted in red.

**FIGURE 10:**
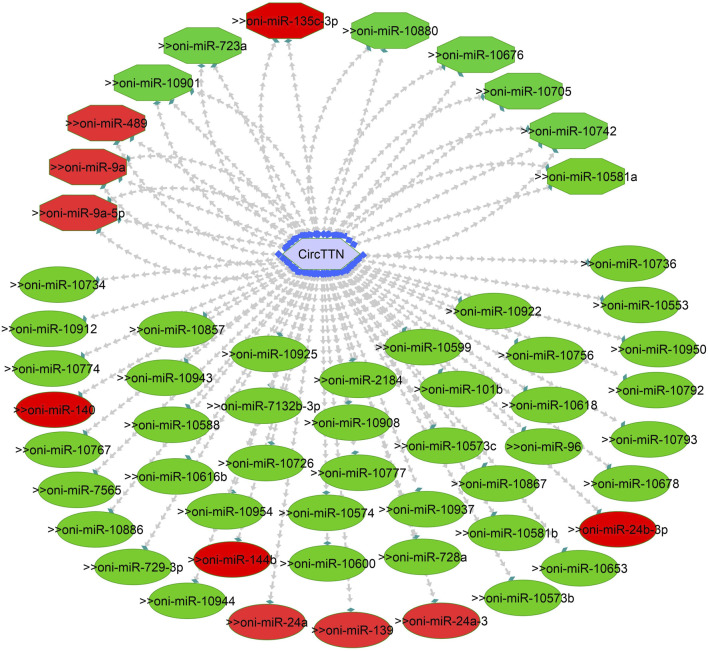
CircTTN-miRNA interaction network regulating the plasticity of muscle growth. Light blue hexagonal nodes represent circRNAs, and circles (single interaction) and octagonal nodes (multiple interactions) represent miRNAs. The potential circRNA-miRNA interactions that could be involved in thermal plasticity of muscle growth are highlighted in red.

**FIGURE 11:**
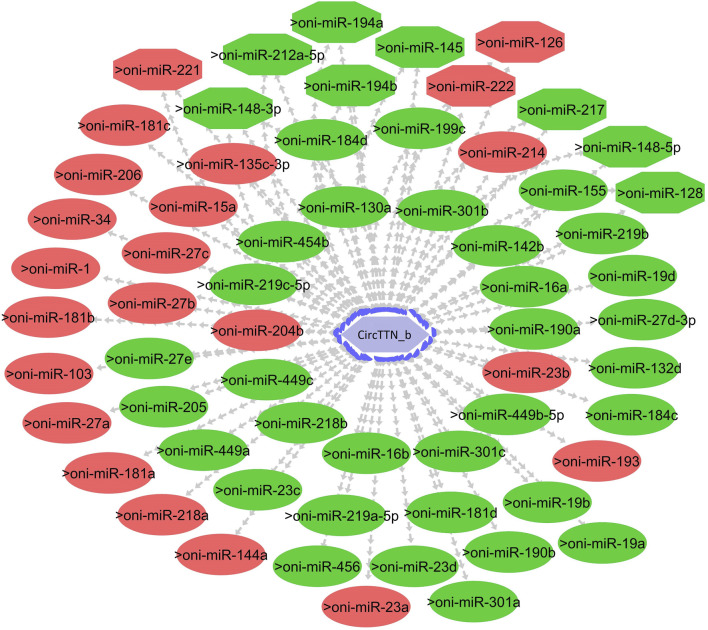
CircTTN_b-miRNA interaction network regulating the plasticity of muscle growth. Light blue hexagonal nodes represent circRNAs, and circles (single interaction) and octagonal nodes (multiple interactions) represent miRNAs. The potential circRNA-miRNA interactions that could be involved in thermal plasticity of muscle growth are highlighted in red.

CircTTN, which is downregulated in the 24°C group compared to 28°C and 32°C counterparts, has a total of 52 miRNA interactions with 50 miRNAs. On the other hand, circTTN_b has 369 interactions with 261 miRNAs. These circRNA-miRNA networks include several important miRNAs related to muscle growth and development, as illustrated in Figures 9–11.

## Discussion

The effect of developmental temperature on circRNA expression and their potential role on growth plasticity are completely unknown. This study revealed clear differences in circRNA expression in fast muscle of juvenile Nile tilapia and concomitant long-term effects on their growth potential when embryos were subjected to 24°C, 28°C, and 32°C temperature. Additionally, we identified 5,141 unique circRNAs across these temperature groups, with 1,604, 1,531, and 1,169 circRNAs exclusively found in the 24°C, 28°C, and 32°C groups, respectively. Notably, three circRNAs (circNexn, circTTN, circTTN_b) exhibited expression plasticity among temperature groups, suggesting a significant role for these circRNAs, in conjunction with temperature, in facilitating long-term growth plasticity.

Several studies have demonstrated the significant impact of temperature during embryonic and larval stages on muscle development and growth, with species-specific responses affecting muscle fiber size and myofibrillar organization (Campos et al., 2013b; Bizuayehu et al., 2015; Aiello et al., 2018; Zhang et al., 2018; Keenan and Currie, 2019; Wragg et al., 2020). For example, Salomão et al. (2018) reported a higher frequency of fibers with larger diameters in Nile tilapia at 22°C compared to 28°C and 30°C, suggesting that growth is primarily driven by hypertrophy at lower temperatures. In contrast, an opposite trend was observed in pacu (*Piaractus mesopotamicus*), where muscle fiber frequency in the >25 μm class was significantly higher at 24°C, while the <50 μm class was lower at this temperature. In our study, Nile tilapia had an enhanced development rate at 32°C, completing the pharyngula stage at 76 h post-fertilization (Table 1). These temporal differences had a significant impact on growth and muscle cellularity, with the 24°C group displaying a higher number of large fibers (larger diameter) at 45 dpf compared to their counterparts in the 28°C and 32°C groups (Figures 1, 2). Conversely, the presence of newly recruited (i.e., small) muscle fibers was more apparent in the 28°C and 32°C groups (Figure 2). Notably, the differences observed among the temperature groups were even more pronounced at 4 months post-fertilization (Figure 2), reflecting the distinct growth mechanisms that were continuously at play. Lower temperatures, as experienced by the 24°C group, may primarily induce hypertrophic growth, characterized by the enlargement of existing muscle fibers. In contrast, higher temperatures, as observed in the 28°C and 32°C groups, may predominantly stimulate hyperplasia, involving the generation of entirely new muscle fibers.

During embryonic and post-embryonic development, gene expression, regulatory RNAs, and epigenetic modulators, such as temperature, play pivotal roles in shaping muscle growth patterns in fish (Wu et al., 2011; Mommens et al., 2014; Liu et al., 2022; Saito et al., 2022). Within this complex framework, miRNAs and circRNAs have emerged as key players capable of modulating gene expression and potentially contributing to the observed effects of temperature on fish growth. Notably, circRNAs are highly abundant during embryonic development, with Liu et al. (2019) identifying 1,028 differentially expressed circRNAs in zebrafish embryos across various developmental stages, underscoring their regulatory significance in embryonic processes.

Comprehensive analysis of high-throughput sequencing data enabled us to discover 5,141 unique circRNAs across all three temperature groups (24°C, 28°C, and 32°C). Our splicing signal analysis revealed that non-GT/AG signaling is the dominant splicing pattern of circRNAs in Nile tilapia muscle, similar to what was observed in crucian carp (*Carassius auratus gibelio*), showing a prominent GU–AG splicing signal (Hu et al., 2019). Interestingly, we also found a high proportion of antisense circRNAs (46.5%) in Nile tilapia muscle, which appears to be a species-specific feature, as no antisense circRNAs have been reported in other fishes. Nevertheless, antisense circRNAs have been documented in other species across various tissues and conditions (Li et al., 2018; Ren et al., 2023). For example, Ma et al. (2021) have identified 209 antisense circRNAs in breast cancer specimens and showed their role in blocking pre-mRNA splicing and translation of mature mRNA.

Among all the unique circRNAs, only 285 circRNAs were common to all temperature groups, while 1,604, 1,531, and 1,169 circRNAs were specific to the 24°C, 28°C, and 32°C groups, respectively. These results suggest that temperature plays a critical role in regulating circRNA expression. Notably, the number of circRNAs in the 24°C group was higher than in the 28°C and 32°C groups. This substantial change in the circRNA transcriptome highlights the possible requirement for a higher number of unique circRNAs in maintaining physiological homeostasis and normal growth at low temperatures. Previous studies in fish, rats, and plants have shown the influence of temperature on circRNA expression (Fan B. et al., 2019; Quan et al., 2021; Liu et al., 2022). For example, Quan et al. (2021) identified 4,138 circRNAs in rainbow trout under high temperatures, and some of them are predicted to regulate the expression of critical genes such as *hsp90ab1, hspa13,* and *hsp70* to adapt to changes in their environment. Therefore, we speculate that the temperature-dependent circRNAs identified in our study might play significant roles in regulating the adaptation and growth plasticity observed in Nile tilapia in response to temperature changes.

It is worth noting that circRNA functions are often linked to their host genes. Recent studies in vertebrates have suggested that many circRNAs may have similar functions to their source genes (Fan L. et al., 2019; Ling et al., 2020). Therefore, we conducted GO and KEGG analyses of the host genes of temperature-specific circRNAs. A number of functional terms, including developmental process, metabolic process, muscle structure development, muscle cell development, and transcription factor binding, were significantly enriched (Figures 6–8). As expected, a functionally arranged network of GO terms visualizes several biological and molecular processes, from embryo development to muscle growth, that are connected with common circRNA-producing genes. These data suggest the potential impact of circRNAs on underlying muscle development and regeneration in Nile tilapia. In addition, KEGG analysis revealed that ECM-receptor interaction pathway, apelin signaling pathway, and focal adhesion were highly correlated with the functions of growth and development (Figures 6–8). Previous studies have shown that focal adhesion pathway is mediated by a composite network of proteins and plays an influential role in the regulation of cell behavior, including cell proliferation, migration, and differentiation (Zhao and Guan, 2011; Ohashi et al., 2017).

By comparing the expression of all unique circRNAs, we identified three circRNAs that were differentially expressed with developmental temperature. Among them, circNexn exhibited a 1.7- og_2_fold up-regulation in the 24°C group and a 1.3-log_2_fold up-regulation in the 32°C group when compared to the 28°C group (Table 3). On the other hand, circTTN and circTTN_b were downregulated in the 28°C and 32°C groups compared to their 24°C counterpart, respectively. CircNexn is derived from the *nexilin* gene, which encodes a protein that binds to *F-actin* and is involved in cell motility. Previous studies have demonstrated that overexpression of *nexilin* promotes cell migration and adhesion and acts in a positive-feedback cycle with *pi3k* and *F-actin* to maintain normal muscle development (Wang et al., 2005; Mei et al., 2009). Thus, our finding of upregulated expression of circNexn suggests its role in compensatory mechanisms to maintain normal muscle function despite temperature change.

In addition, we identified two different circRNAs (circTTN and circTTN_b) produced from the *titin* gene (*LOC100702396*), which were differentially expressed between the 28°C vs. 24°C and 32°C vs. 24°C groups. *Titin* is one of the largest genes in vertebrates and is known to modulate muscle hypertrophy through mechanosensing (van der Pijl et al., 2018). Recent research has also shown that *titin* regulates thin and thick filament formation in muscle (Hessel et al., 2022). Furthermore, circRNA (cTTN1) produced from *titin* has been shown to be essential for normal splicing of the gene *serine and arginine-rich splicing factor 10* (*srsf10*). *Srsf10* is a muscle-specific gene, and its knockdown impairs myoblast differentiation (Wei et al., 2015). Myoblast differentiation is a crucial process for muscle enlargement, playing a fundamental role in increasing the size of existing muscle fibers. During myoblast differentiation in post-embryonic growth, myoblasts fuse together to form multinucleated muscle fibers, and these fibers then increase in size by accumulating more contractile proteins and cellular components (Johnston et al., 2011). Thus, cirRNAs (circTTN and circTTN_b) produced from the *titin* gene and their downregulation in the 24°C group could have an impact on the hypertrophic growth observed in fish muscle.

CircRNAs primarily regulate muscle growth by acting as miRNA sponges. For instance, in snout seabream, competing endogenous networks of circRNA, miRNA and mRNA have been shown to modulate growth (Liu et al., 2021). Additionally, recent research on Nile tilapia indicated that circMef2c, which possesses binding sites for multiple miRNAs, is differentially expressed in relation to growth rate and promotes muscle growth (Rbbani et al., 2023). In the current study, we identified a total of 314 miRNAs that could potentially bind to circNexn, circTTN, and circTTN_b. Among the putative miRNA targets, miR-1, miR-7, miR-22a/b/c, miR-27, miR-181, miR-140, miR-144b, miR-181a/b/c, miR-204b, miR-206, miR-218a, miR-214, miR-221 and miR-449a are particularly important as they have substantial effect on skeletal or cardiac muscle in various fish species including Nile tialpia (Huang et al., 2012; Bizuayehu and Babiak, 2014). In addition, temperature-dependent expression of miR-7a, miR-22, miR-24-3p, miR-27a-3p, miR-27d, miR-34c, miR-130b, miR-181a-3p, miR-204, and miR-449 has been observed in Atlantic cod (*Gadus morhua*), Senegalese sole (*Solea senegalensis*), zebrafish, common carp (*Cyprinus carpio*), and Nile tilapia (Campos et al., 2014a; Bizuayehu et al., 2015; Blödorn et al., 2021). For instance, in Senegalese sole, early developmental temperature exerts a significant influence on miRNA expression, subsequently impacting muscle growth dynamics. Notably, at a temperature of 21 °C, elevated levels of miR-181-5p and miR-206-3p were observed, along with decreased miR-181a-3p levels, potentially promoting growth (Campos et al., 2014b). Additionally, Johnston et al. (2009) reported that miR-181c, miR-1, and miR-206 play significant roles in fiber type switching when zebrafish are exposed to variable developmental temperatures. In common carp, cold temperatures led to the upregulation of miR-27a-3p and miR-27d, resulting in decreased expression of *cdipt* and *mtor* genes (Sun et al., 2019).

MiR-206, miR-1, miR-7, and miR-27b are known to play crucial roles in myoblast differentiation and myocyte fusion by inhibiting specific mRNAs, including *pax3, pax7, pola1*, and *cx43* (Anderson et al., 2006; Ultimo et al., 2018). During myogenesis, increased *myod* expression and decreased *pax3/pax7* expression are achieved through the action of miR-206, miR-1, miR-7, and miR-27b, further influencing muscle differentiation. Additionally, the miR-181 family targets genes such as *hox-a11*, a negative regulator of MYOD, thereby promoting myoblast differentiation and hypertrophic growth (Naguibneva et al., 2006). Moreover, miR-221 and miR-24 contribute to myocyte differentiation and regeneration through the regulation of *myogenin* and *hmga1* (Zacharewicz et al., 2013; Dey et al., 2022). Conversely, miR-489 helps maintain satellite cell quiescence by inhibiting the translation of *myf5*, a critical factor for satellite cell proliferation (Kirby and McCarthy, 2013). Thus, the identification of circNexn, circTTN, and circTTN_b as potential binders for these miRNAs suggests shared roles in sequestering miRNAs and regulating post-transcriptional gene expression. This modulation could enhance energy allocation for long-term growth, particularly under varying developmental temperatures.

In summary, our study undertook a systematic identification and characterization of circRNAs in the fast muscle of Nile tilapia, with the aim of investigating the long-term effects of embryonic temperature on their expression. Our results provide novel evidence of temperature-dependent regulation of circRNA expression in muscle tissue, which may significantly impact development and muscle plasticity. Notably, all differentially expressed circRNAs are derived from muscle-related genes. Furthermore, we identified multiple muscle-associated miRNAs that interact with differentially expressed circRNAs, including circNexn, circTTN, and circTTN_b. These findings collectively expand our knowledge of the molecular mechanisms underlying muscle growth plasticity in response to thermal variation during early ontogeny. To enhance the robustness of our findings and deepen our understanding of the causal relationship between temperature, circRNA expression, and muscle development in Nile tilapia, future investigations could incorporate advanced techniques such as fluorescence *in situ* hybridization (FISH), Northern blotting, and overexpression or knockdown experiments targeting specific circular RNAs and their putative miRNA targets. By integrating these complementary approaches, we can gain a more comprehensive understanding of how temperature-regulated circRNA expression influences the intricate processes of muscle development in Nile tilapia.

## Data Availability

The datasets presented in this study can be found in online repositories. The names of the repository/repositories and accession number(s) can be found here: NCBI (Bioproject EPIFISH, reference PRJNA1028134), SRA accession number SRX24367570-89.
