# Erratum: Embryonic temperature has long-term effects on muscle circRNA expression and somatic growth in Nile tilapia

**DOI:** 10.3389/fcell.2024.1534789

**Published:** 2024-12-05

**Authors:** Frontiers Production Office

**Affiliations:** Frontiers Media SA, Lausanne, Switzerland

**Keywords:** developmental plasticity, thermal plasticity, non-coding RNAs, myogenesis, somatic growth, aquaculture

Due to a production error, the contribution of **Author** “Jorge Galindo-Villegas” was erroneously omitted. The corrected **Author Contributions** Statement appears below.

“GR: Conceptualization, Data curation, Formal Analysis, Investigation, Methodology, Software, Validation, Visualization, Writing–original draft, Writing–review and editing. RM: Data curation, Formal Analysis, Visualization, Writing–review and editing. PS: Data curation, Formal Analysis, Validation, Writing–review and editing, Methodology, Visualization. FS: Data curation, Formal Analysis, Writing–review and editing. AN: Conceptualization, Supervision, Writing–review and editing. RJ: Writing–review and editing, Conceptualization. JG-V: Conceptualization, Supervision, Writing–review and editing. JR: Conceptualization, Supervision, Writing–review and editing. JF: Conceptualization, Funding acquisition, Project administration, Resources, Supervision, Writing–review and editing.”

The publisher apologizes for this mistake. The original version of this article has been updated.

