# Supplementary material for: Embryonic temperature has long-term effects on muscle circRNA expression and somatic growth in Nile tilapia
Source: Front Cell Dev Biol. 2024 Aug 1;12:1369758. doi: 10.3389/fcell.2024.1369758 (PMC11324953; doi:10.3389/fcell.2024.1369758)
Supplement: Supplementary file 1 [file DataSheet1.PDF]

Supplementary Material

Embryonic temperature has long-term effects on muscle circRNA expression and somatic growth in Nile Tilapia

Golam Rbbani[1*], Riaz Murshed[1], Prabhugouda Siriyappagouder[1], Fedor Sharko[2,3], Artem Nedoluzhko[2], Rajesh Joshi[4], Jorge Galindo-Villegas[1], Joost A. M. Raeymaekers[1], Jorge M.O. Fernandes[1,5]*

1 Genomics Division, Faculty of Biosciences and Aquaculture, Nord University, 8049 Bodø, Norway

2 Paleogenomics laboratory, European University at Saint Petersburg, 191187 Saint-Petersburg

3 Paleogenomics laboratory, National Research Center "Kurchatov Institute", 123182 Moscow, Russia

4 GenoMar Genetics AS, 0252 Oslo, Norway

5 Institute of Marine Sciences, Spanish National Research Council, 08003 Barcelona, Spain

* Corresponding author

Email: jorge.m.fernandes@nord.no

Email: baurumon@gmail.com

Supplementary Tables

Table S1. Water quality parameters (dissolved oxygen, pH, salinity, nitrates, nitrites and ammonia) throughout the experimental period.

Water quality measurement									
Day		Day 0	Day 1	Day 2	Day 3	Day 4	Day 5	Day 6	Day 7
Photoperiod		13:11	13:11	13:11	13:11	13:11	13:11	13:11	13:11
Temperature group		6.8	6.65	6.8	6.8				
Dissolved O_2 (mg/L)	32 ˚C								
	28 ˚C	7.2	7.16	7.5	7.2	6.4			
	24 ˚C	7.7	7.62	7.7	7.6	7.7	7.5	7.7	7.6
pH	32 ˚C	7.53	7.95	7.96	7.99				
	28 ˚C	7.66	8.01	7.88	7.92	8.07			
	24 ˚C	7.88	7.85	7.92	8.01	8.05	7.93	7.95	7.98
Salinity (gm/L)	32 ˚C	2.0-2.5	2.0-2.5	2.0-2.5	2.0-2.5				
	28 ˚C	2.0-2.5	2.0-2.5	2.0-2.5	2.0-2.5	2.0-2.5	2.0-2.5	2.0-2.5	
	24 ˚C	2.0-2.5	2.0-2.5	2.0-2.5	2.0-2.5	2.0-2.5	2.0-2.5	2.0-2.5	2.0-2.5
Nitrates*	32 ˚C	0			0				0
	28 ˚C	0			0				0
	24 ˚C	0			0				0
Nitrites*	32 ˚C	0			0				0
	28 ˚C	0			0				0
	24 ˚C	0			0				0
Ammonia*	32 ˚C	0.018			0.4				0
	28 ˚C	0.011			0.42				0
	24 ˚C	0.021			0.42				0.024

* Nitrate, nitrites and ammonia were analyzed thrice during the experimental period since we changed half of the tank water daily.

Table S2. Fertilization, hatching and survival data of Nile tilapia embryos incubated at different temperatures.

Temperature Group	Replicate	Total eggs	Unfertilized eggs	Fertilized eggs	Survived and hatched out larvae	Total surviving larvae	Survival rate of hatched larvae (%)
**32 ˚C**	Rep 1	230	26	204	135	113	83.7
	Rep 2	230	24	206	129	122	94.51
	Rep 3	230	20	210	127	116	91.3
**28 ˚C**	Rep 1	230	22	208	140	128	91.4
	Rep 2	230	32	198	145	131	90.3
	Rep 3	230	25	205	148	133	89.8
**24 ˚C**	Rep 1	230	22	208	147	137	93.2
	Rep 2	230	31	199	151	138	91.4
	Rep 3	230	40	190	153	136	88.9

Table S3. Linear covariance analysis of weight at 45 dpf and 4-month post-fertilization in Nile tilapia treated at different developmental temperatures

Variable	Estimate	Std.Error	t.value	Pr(>|t|)
(Intercept)	168.325709	15.014089	11.2111834	0.001521301
Mean_Weight_at_45dpf	14.856659	3.992753	3.7209061	0.033786009
Temperature 28 ˚C	9.816635	2.624454	3.7404483	0.033334283
Temperature 32 ˚C	43.953347	2.849955	15.4224682	0.000592209
ReplicateR2	2.131529	2.668779	0.7986907	0.482853098
ReplicateR3	-21.711247	2.683316	-8.0912015	0.003945039

Table S4. Summary of RNA sequencing and mapping statistics of circRNAs in Nile tilapia fast muscle.

Temperature groups	Sample ID	Number of raw reads (millions)	Number of clean reads (after fastp)	Number of mapped reads	Mapping efficiency to the reference genome (%)
24 °C	24_1	143.32	140.65	143.10	99.23
	24_2	106.83	105.80	107.10	99.56
	24_3	180.70	172.56	174.58	99.58
	24_4	94.06	92.16	93.39	99.2
	24_6	109.11	97.51	97.18	99.66
	24_7	251.26	239.40	238.93	97.46
	24_8	170.72	168.05	169.94	99.36
	24_9	97.13	95.86	95.25	99.37
28 °C	28_1	260.00	244.82	240.25	98.18
	28_2	134.02	132.09	131.09	99.25
	28_3	227.85	221.57	219.16	98.91
	28_4	132.86	124.83	123.37	98.83
	28_5	156.29	153.23	152.11	99.27
	28_7	143.05	139.93	138.99	99.33
	28_8	196.58	187.37	185.27	98.88
	28_9	85.26	84.30	83.86	99.49
32 °C	32_1	267.66	260.22	256.33	98.51
	32_2	272.28	261.90	257.29	98.24
	32_3	245.53	242.78	240.77	99.17
	32_5	175.09	170.07	167.51	98.5
	32_6	156.79	155.52	154.85	99.57
	32_7	140.74	137.53	136.33	99.13
	32_8	158.21	150.66	149.57	99.28
	32_9	103.02	102.02	101.53	99.52

N.B.: Samples 24_5, 28_5 and 32_4 *had a considerably lower sequencing output than the rest. Thus, they were excluded from further analysis.*

Table S5. Differentially expressed circRNAs and their possible miRNA targets

Circular RNA	Targeted miRNA	Energy score (Kcal/Mol)
CircNexn	oni-miR-10577	144
CircNexn	oni-miR-10578	157
CircNexn	oni-miR-10594c	145
CircNexn	oni-miR-10605	141
CircNexn	oni-miR-10618	143
CircNexn	oni-miR-10623	158
CircNexn	oni-miR-10637	147
CircNexn	oni-miR-10638	148
CircNexn	oni-miR-10640	146
CircNexn	oni-miR-10660	173
CircNexn	oni-miR-10670	145
CircNexn	oni-miR-10686	145
CircNexn	oni-miR-10688	153
CircNexn	oni-miR-10691	149
CircNexn	oni-miR-10699	150
CircNexn	oni-miR-10705	146
CircNexn	oni-miR-10717	142
CircNexn	oni-miR-10719	147
CircNexn	oni-miR-10724	146
CircNexn	oni-miR-10734	149
CircNexn	oni-miR-10753	143
CircNexn	oni-miR-10755	152
CircNexn	oni-miR-10757	157
CircNexn	oni-miR-10759	148
CircNexn	oni-miR-10759	156
CircNexn	oni-miR-10761	164
CircNexn	oni-miR-10770	155
CircNexn	oni-miR-10772	163

CircNexn	oni-miR-10775	148
CircNexn	oni-miR-10803	147
CircNexn	oni-miR-10803	152
CircNexn	oni-miR-10829	151
CircNexn	oni-miR-10854	164
CircNexn	oni-miR-10857	168
CircNexn	oni-miR-10899	152
CircNexn	oni-miR-10913	148
CircNexn	oni-miR-10915	158
CircNexn	oni-miR-10925	147
CircNexn	oni-miR-10938	144
CircNexn	oni-miR-10977	140
CircNexn	oni-miR-132d	141
CircNexn	oni-miR-190b	151
CircNexn	oni-miR-210	162
CircNexn	oni-miR-22a	140
CircNexn	oni-miR-22b	140
CircNexn	oni-miR-22c	140
CircNexn	oni-miR-27d-5p	149
CircNexn	oni-miR-34	143
CircNexn	oni-miR-449a	147
CircNexn	oni-miR-449b-5p	140
CircNexn	oni-miR-449c	141
CircNexn	oni-miR-7	159
CircTTN	oni-miR-101b	149
CircTTN	oni-miR-10553	143
CircTTN	oni-miR-10573b	149
CircTTN	oni-miR-10573c	153
CircTTN	oni-miR-10574	159
CircTTN	oni-miR-10581a	151

CircTTN	oni-miR-10581a	160
CircTTN	oni-miR-10581b	140
CircTTN	oni-miR-10588	149
CircTTN	oni-miR-10599	145
CircTTN	oni-miR-10600	161
CircTTN	oni-miR-10616b	154
CircTTN	oni-miR-10618	152
CircTTN	oni-miR-10653	151
CircTTN	oni-miR-10676	146
CircTTN	oni-miR-10676	154
CircTTN	oni-miR-10678	145
CircTTN	oni-miR-10705	146
CircTTN	oni-miR-10705	147
CircTTN	oni-miR-10726	150
CircTTN	oni-miR-10734	140
CircTTN	oni-miR-10736	145
CircTTN	oni-miR-10742	146
CircTTN	oni-miR-10742	151
CircTTN	oni-miR-10742	160
CircTTN	oni-miR-10756	158
CircTTN	oni-miR-10767	140
CircTTN	oni-miR-10774	142
CircTTN	oni-miR-10777	158
CircTTN	oni-miR-10792	144
CircTTN	oni-miR-10793	147
CircTTN	oni-miR-10857	142
CircTTN	oni-miR-10867	152
CircTTN	oni-miR-10880	146
CircTTN	oni-miR-10880	159
CircTTN	oni-miR-10886	146

CircTTN	oni-miR-10901	145
CircTTN	oni-miR-10901	148
CircTTN	oni-miR-10901	148
CircTTN	oni-miR-10908	158
CircTTN	oni-miR-10912	140
CircTTN	oni-miR-10922	148
CircTTN	oni-miR-10925	143
CircTTN	oni-miR-10937	150
CircTTN	oni-miR-10943	145
CircTTN	oni-miR-10944	146
CircTTN	oni-miR-10950	142
CircTTN	oni-miR-10954	150
CircTTN	oni-miR-135c-3p	141
CircTTN	oni-miR-135c-3p	152
CircTTN	oni-miR-139	155
CircTTN	oni-miR-140	141
CircTTN	oni-miR-144b	159
CircTTN	oni-miR-2184	149
CircTTN	oni-miR-24a	148
CircTTN	oni-miR-24a-3	148
CircTTN	oni-miR-24b-3p	148
CircTTN	oni-miR-489	140
CircTTN	oni-miR-489	142
CircTTN	oni-miR-7132b-3p	150
CircTTN	oni-miR-723a	144
CircTTN	oni-miR-723a	153
CircTTN	oni-miR-728a	162
CircTTN	oni-miR-729-3p	151
CircTTN	oni-miR-7565	142
CircTTN	oni-miR-96	154

CircTTN	oni-miR-9a	151
CircTTN	oni-miR-9a	151
CircTTN	oni-miR-9a	153
CircTTN	oni-miR-9a-5p	151
CircTTN	oni-miR-9a-5p	151
CircTTN	oni-miR-9a-5p	153
CircTTN_b	oni-miR-1	140
CircTTN_b	oni-miR-103	151
CircTTN_b	oni-miR-10551	149
CircTTN_b	oni-miR-10552	150
CircTTN_b	oni-miR-10552	150
CircTTN_b	oni-miR-10556a	146
CircTTN_b	oni-miR-10556b	153
CircTTN_b	oni-miR-10559	160
CircTTN_b	oni-miR-10563	154
CircTTN_b	oni-miR-10573b	140
CircTTN_b	oni-miR-10573b	140
CircTTN_b	oni-miR-10573c	140
CircTTN_b	oni-miR-10573c	140
CircTTN_b	oni-miR-10577	155
CircTTN_b	oni-miR-10581a	149
CircTTN_b	oni-miR-10581a	156
CircTTN_b	oni-miR-10581b	154
CircTTN_b	oni-miR-10581b	154
CircTTN_b	oni-miR-10583	146
CircTTN_b	oni-miR-10584	163
CircTTN_b	oni-miR-10589	152
CircTTN_b	oni-miR-10589	156
CircTTN_b	oni-miR-10591	161
CircTTN_b	oni-miR-10595	153

CircTTN_b	oni-miR-10596-3p	170
CircTTN_b	oni-miR-10597a	153
CircTTN_b	oni-miR-10597b	156
CircTTN_b	oni-miR-10597c	153
CircTTN_b	oni-miR-10597d	160
CircTTN_b	oni-miR-10599	160
CircTTN_b	oni-miR-10601	159
CircTTN_b	oni-miR-10603a	167
CircTTN_b	oni-miR-10604	146
CircTTN_b	oni-miR-10604	147
CircTTN_b	oni-miR-10605	143
CircTTN_b	oni-miR-10605	145
CircTTN_b	oni-miR-10608b	140
CircTTN_b	oni-miR-10608b	144
CircTTN_b	oni-miR-10610b	157
CircTTN_b	oni-miR-10614	158
CircTTN_b	oni-miR-10615b	148
CircTTN_b	oni-miR-10616a	155
CircTTN_b	oni-miR-10616b	148
CircTTN_b	oni-miR-10620	148
CircTTN_b	oni-miR-10623	140
CircTTN_b	oni-miR-10623	142
CircTTN_b	oni-miR-10623	144
CircTTN_b	oni-miR-10629	144
CircTTN_b	oni-miR-10629	144
CircTTN_b	oni-miR-10630	145
CircTTN_b	oni-miR-10633	147
CircTTN_b	oni-miR-10634	143
CircTTN_b	oni-miR-10637	151
CircTTN_b	oni-miR-10639	152

CircTTN_b	oni-miR-10642	146
CircTTN_b	oni-miR-10642	147
CircTTN_b	oni-miR-10642	158
CircTTN_b	oni-miR-10643	155
CircTTN_b	oni-miR-10643	158
CircTTN_b	oni-miR-10645	163
CircTTN_b	oni-miR-10648	151
CircTTN_b	oni-miR-10650	150
CircTTN_b	oni-miR-10654	144
CircTTN_b	oni-miR-10655	149
CircTTN_b	oni-miR-10655	162
CircTTN_b	oni-miR-10655	164
CircTTN_b	oni-miR-10660	152
CircTTN_b	oni-miR-10660	158
CircTTN_b	oni-miR-10660	160
CircTTN_b	oni-miR-10663	154
CircTTN_b	oni-miR-10665	154
CircTTN_b	oni-miR-10668	163
CircTTN_b	oni-miR-10670	162
CircTTN_b	oni-miR-10672	151
CircTTN_b	oni-miR-10675	149
CircTTN_b	oni-miR-10676	146
CircTTN_b	oni-miR-10676	153
CircTTN_b	oni-miR-10677	149
CircTTN_b	oni-miR-10677	151
CircTTN_b	oni-miR-10677	154
CircTTN_b	oni-miR-10678	146
CircTTN_b	oni-miR-10681	142
CircTTN_b	oni-miR-10688	146
CircTTN_b	oni-miR-10693	140

CircTTN_b	oni-miR-10693	140
CircTTN_b	oni-miR-10693	142
CircTTN_b	oni-miR-10698	140
CircTTN_b	oni-miR-10698	164
CircTTN_b	oni-miR-107	150
CircTTN_b	oni-miR-10702	159
CircTTN_b	oni-miR-10703	140
CircTTN_b	oni-miR-10703	145
CircTTN_b	oni-miR-10708	143
CircTTN_b	oni-miR-10711	143
CircTTN_b	oni-miR-10713	152
CircTTN_b	oni-miR-10715	145
CircTTN_b	oni-miR-10715	150
CircTTN_b	oni-miR-10719	148
CircTTN_b	oni-miR-10721	153
CircTTN_b	oni-miR-10721	167
CircTTN_b	oni-miR-10723	145
CircTTN_b	oni-miR-10723	151
CircTTN_b	oni-miR-10725	150
CircTTN_b	oni-miR-10726	147
CircTTN_b	oni-miR-10726	147
CircTTN_b	oni-miR-10727	140
CircTTN_b	oni-miR-10730	144
CircTTN_b	oni-miR-10730	152
CircTTN_b	oni-miR-10731	148
CircTTN_b	oni-miR-10732	145
CircTTN_b	oni-miR-10734	148
CircTTN_b	oni-miR-10734	152
CircTTN_b	oni-miR-10742	142
CircTTN_b	oni-miR-10742	147

CircTTN_b	oni-miR-10742	156
CircTTN_b	oni-miR-10742	180
CircTTN_b	oni-miR-10745	158
CircTTN_b	oni-miR-10745	159
CircTTN_b	oni-miR-10748	140
CircTTN_b	oni-miR-10748	170
CircTTN_b	oni-miR-10752	140
CircTTN_b	oni-miR-10753	143
CircTTN_b	oni-miR-10757	145
CircTTN_b	oni-miR-10761	150
CircTTN_b	oni-miR-10761	154
CircTTN_b	oni-miR-10761	163
CircTTN_b	oni-miR-10765	157
CircTTN_b	oni-miR-10768	153
CircTTN_b	oni-miR-10775	140
CircTTN_b	oni-miR-10776	146
CircTTN_b	oni-miR-10779	150
CircTTN_b	oni-miR-10779	157
CircTTN_b	oni-miR-10780	150
CircTTN_b	oni-miR-10780	171
CircTTN_b	oni-miR-10781	140
CircTTN_b	oni-miR-10786	141
CircTTN_b	oni-miR-10787	148
CircTTN_b	oni-miR-10789	168
CircTTN_b	oni-miR-10797	146
CircTTN_b	oni-miR-10797	147
CircTTN_b	oni-miR-10798	144
CircTTN_b	oni-miR-10798	159
CircTTN_b	oni-miR-10805	143
CircTTN_b	oni-miR-10806	164

CircTTN_b	oni-miR-10816	158
CircTTN_b	oni-miR-10821	149
CircTTN_b	oni-miR-10822	141
CircTTN_b	oni-miR-10822	146
CircTTN_b	oni-miR-10824	147
CircTTN_b	oni-miR-10826	142
CircTTN_b	oni-miR-10829	140
CircTTN_b	oni-miR-10833	145
CircTTN_b	oni-miR-10834	155
CircTTN_b	oni-miR-10835	161
CircTTN_b	oni-miR-10835	166
CircTTN_b	oni-miR-10841	144
CircTTN_b	oni-miR-10841	152
CircTTN_b	oni-miR-10842	145
CircTTN_b	oni-miR-10844	155
CircTTN_b	oni-miR-10853	145
CircTTN_b	oni-miR-10854	160
CircTTN_b	oni-miR-10856	140
CircTTN_b	oni-miR-10856	143
CircTTN_b	oni-miR-10858	146
CircTTN_b	oni-miR-10862	147
CircTTN_b	oni-miR-10862	151
CircTTN_b	oni-miR-10868	144
CircTTN_b	oni-miR-10869	141
CircTTN_b	oni-miR-10869	145
CircTTN_b	oni-miR-10869	148
CircTTN_b	oni-miR-10869	152
CircTTN_b	oni-miR-10869	154
CircTTN_b	oni-miR-10869	156
CircTTN_b	oni-miR-10872	154

CircTTN_b	oni-miR-10875	140
CircTTN_b	oni-miR-10875	147
CircTTN_b	oni-miR-10876	140
CircTTN_b	oni-miR-10876	142
CircTTN_b	oni-miR-10876	142
CircTTN_b	oni-miR-10876	148
CircTTN_b	oni-miR-10876	149
CircTTN_b	oni-miR-10878	165
CircTTN_b	oni-miR-10879	149
CircTTN_b	oni-miR-10880	141
CircTTN_b	oni-miR-10880	152
CircTTN_b	oni-miR-10882	159
CircTTN_b	oni-miR-10883	143
CircTTN_b	oni-miR-10887	159
CircTTN_b	oni-miR-10891	147
CircTTN_b	oni-miR-10892	151
CircTTN_b	oni-miR-10894	154
CircTTN_b	oni-miR-10894	160
CircTTN_b	oni-miR-10897	149
CircTTN_b	oni-miR-10897	157
CircTTN_b	oni-miR-10899	154
CircTTN_b	oni-miR-10900b	142
CircTTN_b	oni-miR-10900b	159
CircTTN_b	oni-miR-10901	155
CircTTN_b	oni-miR-10910	145
CircTTN_b	oni-miR-10910	146
CircTTN_b	oni-miR-10910	149
CircTTN_b	oni-miR-10910	152
CircTTN_b	oni-miR-10910	155
CircTTN_b	oni-miR-10910	157

CircTTN_b	oni-miR-10912	148
CircTTN_b	oni-miR-10922	159
CircTTN_b	oni-miR-10926	146
CircTTN_b	oni-miR-10926	155
CircTTN_b	oni-miR-10927	145
CircTTN_b	oni-miR-10927	154
CircTTN_b	oni-miR-10929	143
CircTTN_b	oni-miR-10931	157
CircTTN_b	oni-miR-10932	144
CircTTN_b	oni-miR-10932	145
CircTTN_b	oni-miR-10932	145
CircTTN_b	oni-miR-10933	153
CircTTN_b	oni-miR-10935	159
CircTTN_b	oni-miR-10938	157
CircTTN_b	oni-miR-10941	150
CircTTN_b	oni-miR-10941	158
CircTTN_b	oni-miR-10941	163
CircTTN_b	oni-miR-10942	154
CircTTN_b	oni-miR-10943	155
CircTTN_b	oni-miR-10943	169
CircTTN_b	oni-miR-10944	145
CircTTN_b	oni-miR-10948	140
CircTTN_b	oni-miR-10949	146
CircTTN_b	oni-miR-10953	145
CircTTN_b	oni-miR-10960	148
CircTTN_b	oni-miR-10961	158
CircTTN_b	oni-miR-10962	147
CircTTN_b	oni-miR-10964	155
CircTTN_b	oni-miR-10966	146
CircTTN_b	oni-miR-10966	157

CircTTN_b	oni-miR-10967	149
CircTTN_b	oni-miR-10968	145
CircTTN_b	oni-miR-10968	156
CircTTN_b	oni-miR-10971	140
CircTTN_b	oni-miR-10971	153
CircTTN_b	oni-miR-10973	141
CircTTN_b	oni-miR-10975	153
CircTTN_b	oni-miR-10977	157
CircTTN_b	oni-miR-126	147
CircTTN_b	oni-miR-126	153
CircTTN_b	oni-miR-128	145
CircTTN_b	oni-miR-128	145
CircTTN_b	oni-miR-130a	148
CircTTN_b	oni-miR-130b-3p	149
CircTTN_b	oni-miR-130b-5p	140
CircTTN_b	oni-miR-130b-5p	140
CircTTN_b	oni-miR-130b-5p	149
CircTTN_b	oni-miR-132d	150
CircTTN_b	oni-miR-135c-3p	147
CircTTN_b	oni-miR-142b	152
CircTTN_b	oni-miR-144a	149
CircTTN_b	oni-miR-145	141
CircTTN_b	oni-miR-145	146
CircTTN_b	oni-miR-148-3p	145
CircTTN_b	oni-miR-148-3p	146
CircTTN_b	oni-miR-148-3p	156
CircTTN_b	oni-miR-148-5p	148
CircTTN_b	oni-miR-148-5p	150
CircTTN_b	oni-miR-155	153
CircTTN_b	oni-miR-15a	145

CircTTN_b	oni-miR-16a	167
CircTTN_b	oni-miR-16b	167
CircTTN_b	oni-miR-181a	151
CircTTN_b	oni-miR-181b	148
CircTTN_b	oni-miR-181c	159
CircTTN_b	oni-miR-181d	151
CircTTN_b	oni-miR-184c	168
CircTTN_b	oni-miR-184d	142
CircTTN_b	oni-miR-190a	148
CircTTN_b	oni-miR-190b	151
CircTTN_b	oni-miR-193	141
CircTTN_b	oni-miR-194a	140
CircTTN_b	oni-miR-194a	152
CircTTN_b	oni-miR-194b	144
CircTTN_b	oni-miR-194b	150
CircTTN_b	oni-miR-199c	143
CircTTN_b	oni-miR-19a	147
CircTTN_b	oni-miR-19b	142
CircTTN_b	oni-miR-19d	142
CircTTN_b	oni-miR-204b	155
CircTTN_b	oni-miR-205	141
CircTTN_b	oni-miR-206	140
CircTTN_b	oni-miR-212a-5p	154
CircTTN_b	oni-miR-212a-5p	155
CircTTN_b	oni-miR-214	151
CircTTN_b	oni-miR-216a	154
CircTTN_b	oni-miR-216a	156
CircTTN_b	oni-miR-216b	140
CircTTN_b	oni-miR-217	153
CircTTN_b	oni-miR-217	168

CircTTN_b	oni-miR-218a	151
CircTTN_b	oni-miR-218b	151
CircTTN_b	oni-miR-219a-5p	150
CircTTN_b	oni-miR-219b	150
CircTTN_b	oni-miR-219c-5p	150
CircTTN_b	oni-miR-221	158
CircTTN_b	oni-miR-221	163
CircTTN_b	oni-miR-222	148
CircTTN_b	oni-miR-222	168
CircTTN_b	oni-miR-23a	150
CircTTN_b	oni-miR-23b	150
CircTTN_b	oni-miR-23c	154
CircTTN_b	oni-miR-23d	154
CircTTN_b	oni-miR-24b-5p	146
CircTTN_b	oni-miR-27a	153
CircTTN_b	oni-miR-27b	155
CircTTN_b	oni-miR-27c	161
CircTTN_b	oni-miR-27d-3p	153
CircTTN_b	oni-miR-27e	153
CircTTN_b	oni-miR-301a	144
CircTTN_b	oni-miR-301b	146
CircTTN_b	oni-miR-301c	156
CircTTN_b	oni-miR-3120-3p	140
CircTTN_b	oni-miR-338	147
CircTTN_b	oni-miR-33a-3p	148
CircTTN_b	oni-miR-34	147
CircTTN_b	oni-miR-3553	151
CircTTN_b	oni-miR-429b	151
CircTTN_b	oni-miR-449a	145
CircTTN_b	oni-miR-449b-5p	158

CircTTN_b	oni-miR-449c	156
CircTTN_b	oni-miR-454b	157
CircTTN_b	oni-miR-455	143
CircTTN_b	oni-miR-455	154
CircTTN_b	oni-miR-456	146
CircTTN_b	oni-miR-457	147
CircTTN_b	oni-miR-458	155
CircTTN_b	oni-miR-458	155
CircTTN_b	oni-miR-458	173
CircTTN_b	oni-miR-489	153
CircTTN_b	oni-miR-489	153
CircTTN_b	oni-miR-7	149
CircTTN_b	oni-miR-7	152
CircTTN_b	oni-miR-7132a-5p	156
CircTTN_b	oni-miR-7132b-3p	159
CircTTN_b	oni-miR-7132b-5p	148
CircTTN_b	oni-miR-7147	145
CircTTN_b	oni-miR-723a	150
CircTTN_b	oni-miR-723a	151
CircTTN_b	oni-miR-726b	150
CircTTN_b	oni-miR-727a	142
CircTTN_b	oni-miR-727a	155
CircTTN_b	oni-miR-727b	143
CircTTN_b	oni-miR-727b	151
CircTTN_b	oni-miR-729-5p	142
CircTTN_b	oni-miR-729-5p	146
CircTTN_b	oni-miR-730	149
CircTTN_b	oni-miR-731	148
CircTTN_b	oni-miR-734	145
CircTTN_b	oni-miR-7565	140

CircTTN_b	oni-miR-7565	153
CircTTN_b	oni-miR-7565	168
CircTTN_b	oni-miR-8159	145
CircTTN_b	oni-miR-8160a	152
CircTTN_b	oni-miR-8160a	161
CircTTN_b	oni-miR-8160b	152
CircTTN_b	oni-miR-8160b	161
CircTTN_b	oni-miR-96	141
CircTTN_b	oni-miR-96	162
CircTTN_b	oni-miR-9a	151
CircTTN_b	oni-miR-9a	161
CircTTN_b	oni-miR-9a-3p	156
CircTTN_b	oni-miR-9a-5p	151
CircTTN_b	oni-miR-9a-5p	161
CircTTN_b	oni-miR-9a-7-3p	156

Supplementary Table S6. Morphometric data of the fish used for circRNA transcriptomics.

Embryonic temperature (˚C)	Total length (cm)	Standard length (cm)	Weight (g)
24	6.5	5.4	4.36
	6.7	5.6	4.69
	7	6.1	5.47
	5.3	4.3	2.37
	6	5.1	3.31
	5.7	4.8	2.82
	6.8	5.6	5.15
	5.9	4.8	3.19
	6.6	5.5	4.37
28	7	5.7	5.45
	6.7	5.5	4.66
	5.9	4.9	3.45
	6	5	3.64
	6.8	5.5	5.63
	6.2	5.3	3.96
	6	4.9	3.66
	5.5	4.6	2.63
	5.9	4.9	3.31
32	6.4	5.1	4.2
	6.6	5.3	4.94
	6.9	5.5	5.27
	7.3	6.1	6.6
	6.7	5.5	5.24
	6.1	5	4.38
	5.7	4.7	3.05
	6.5	5.4	4.62
	5.8	4.7	2.98

Supplementary Table S7. Average junction reads of the three significantly differentially expressed circRNAs in each temperature group.

CircRNA name	Embryonic temperature group		
	24 °C	28 °C	32 °C
CircNexn	46	7	52
CircTTN	11	45	18
CircTTN_b	16	30	50

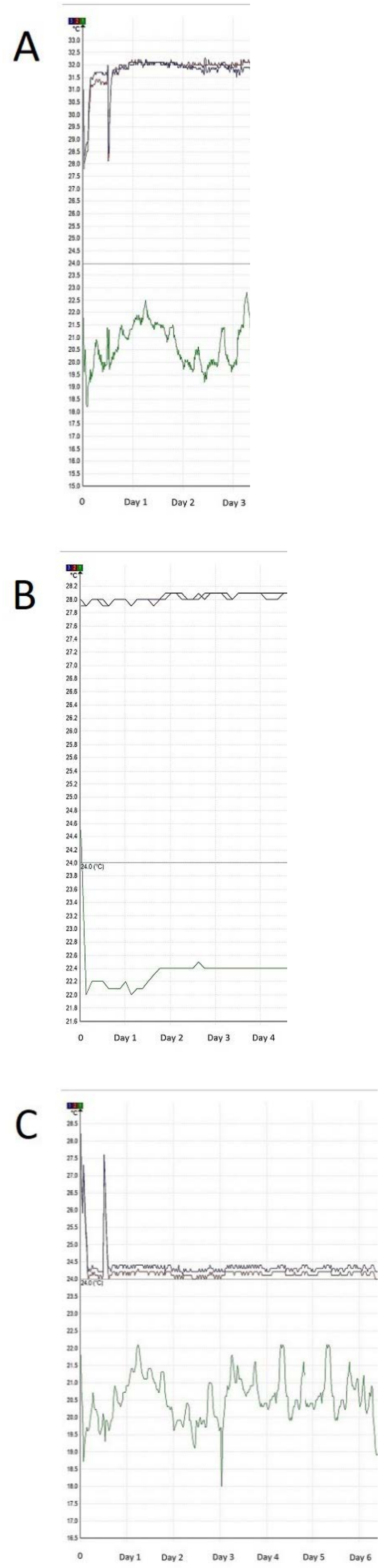

Figure S1. Daily temperature measurements (°C) in the experimental tanks during early development until the pharyngula stage. A), B) and C) display the daily temperature measurements recorded in the 32 °C, 28 °C and 24 °C tanks using an EBI probe.

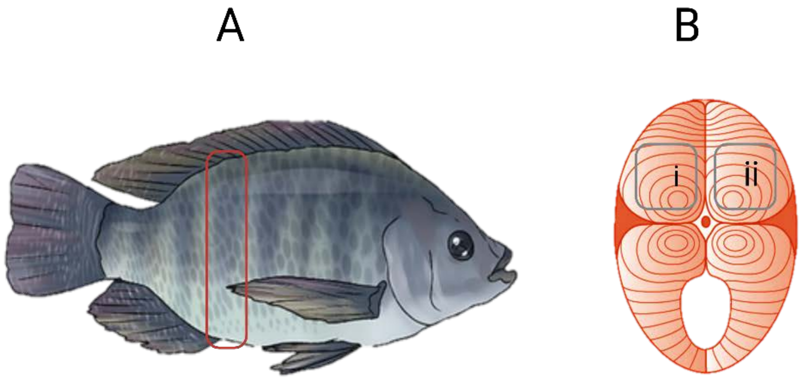

Figure S2. Graphic representation of the fish and the muscle cross-section taken at 45 days post-fertilization. (A) Nile tilapia sampled at 45 days post-fertilization is depicted, with the marked area indicating the region from which muscle samples were obtained. (B) Muscle cross-section showing the marked areas for (i) muscle block used for RNA extraction and (ii) muscle block used for histological analysis

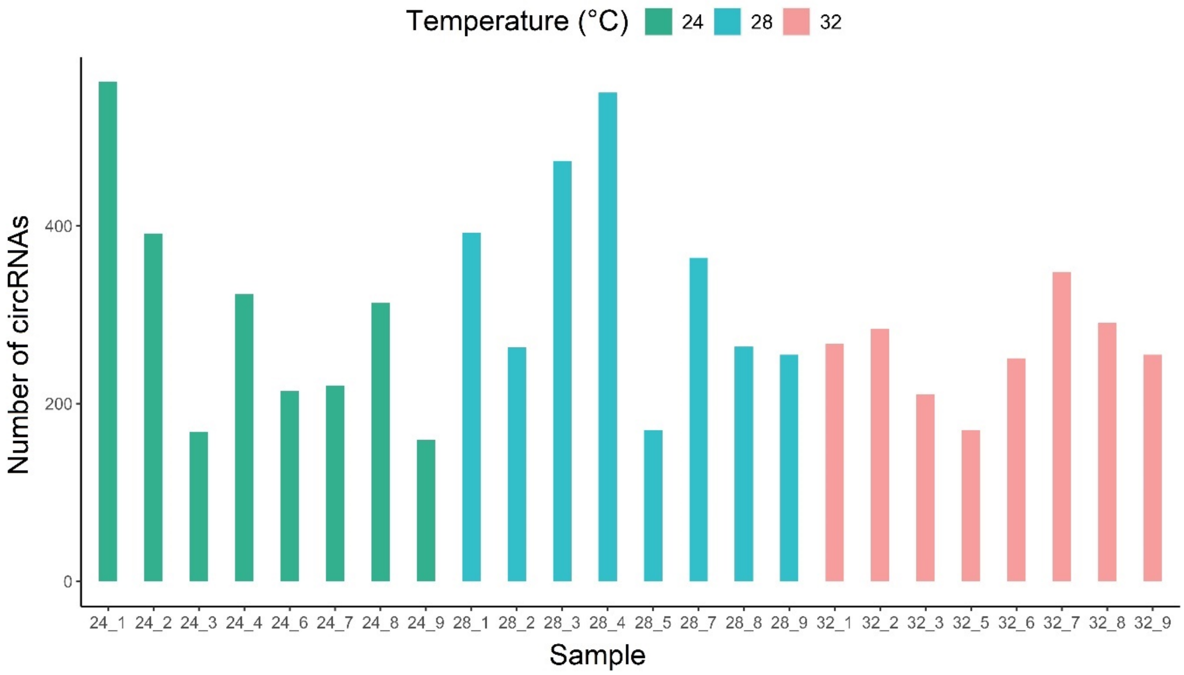

Figure S3. Number of circRNAs identified in the three temperature groups. Green, blue and red bars represent 24 °C, 28 °C and 32 °C groups, respectively.

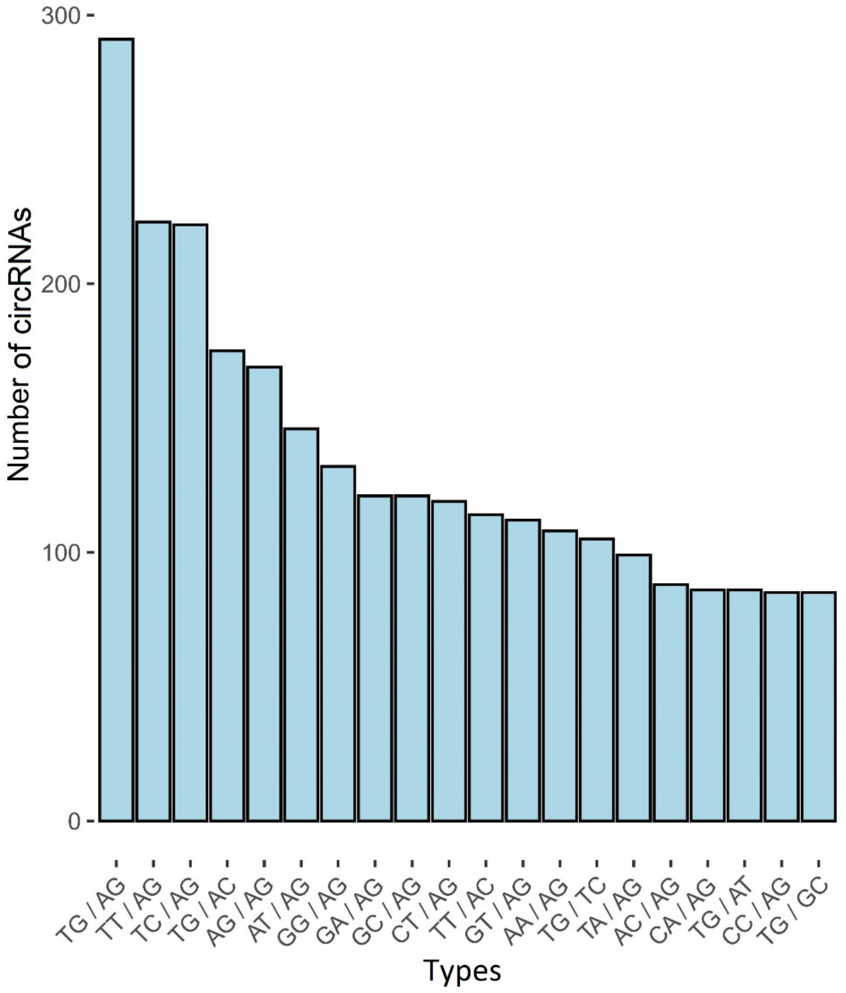

Figure S4. Splice signals within the circRNAs identified in Nile tilapia. The histogram shows the top 20 splice signals in circRNA flanking exons.

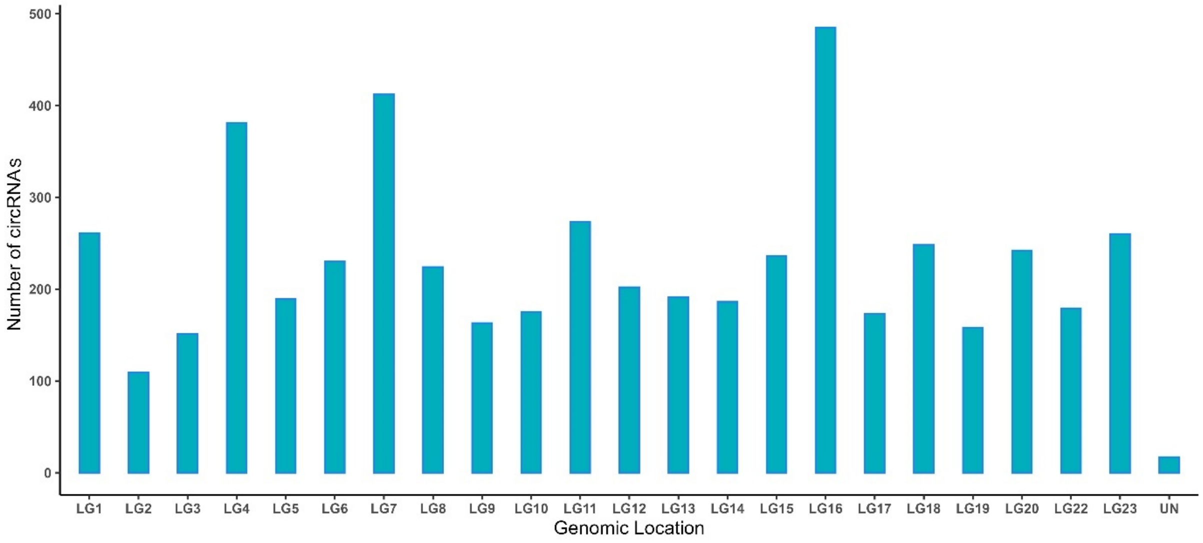

Figure S5. CircRNA distribution throughout the linkage groups of Nile tilapia genome. The x-axis represents the location of the circRNAs in linkage groups (LG) and unknown (UN) positions, while the y-axis shows the number of circRNAs.

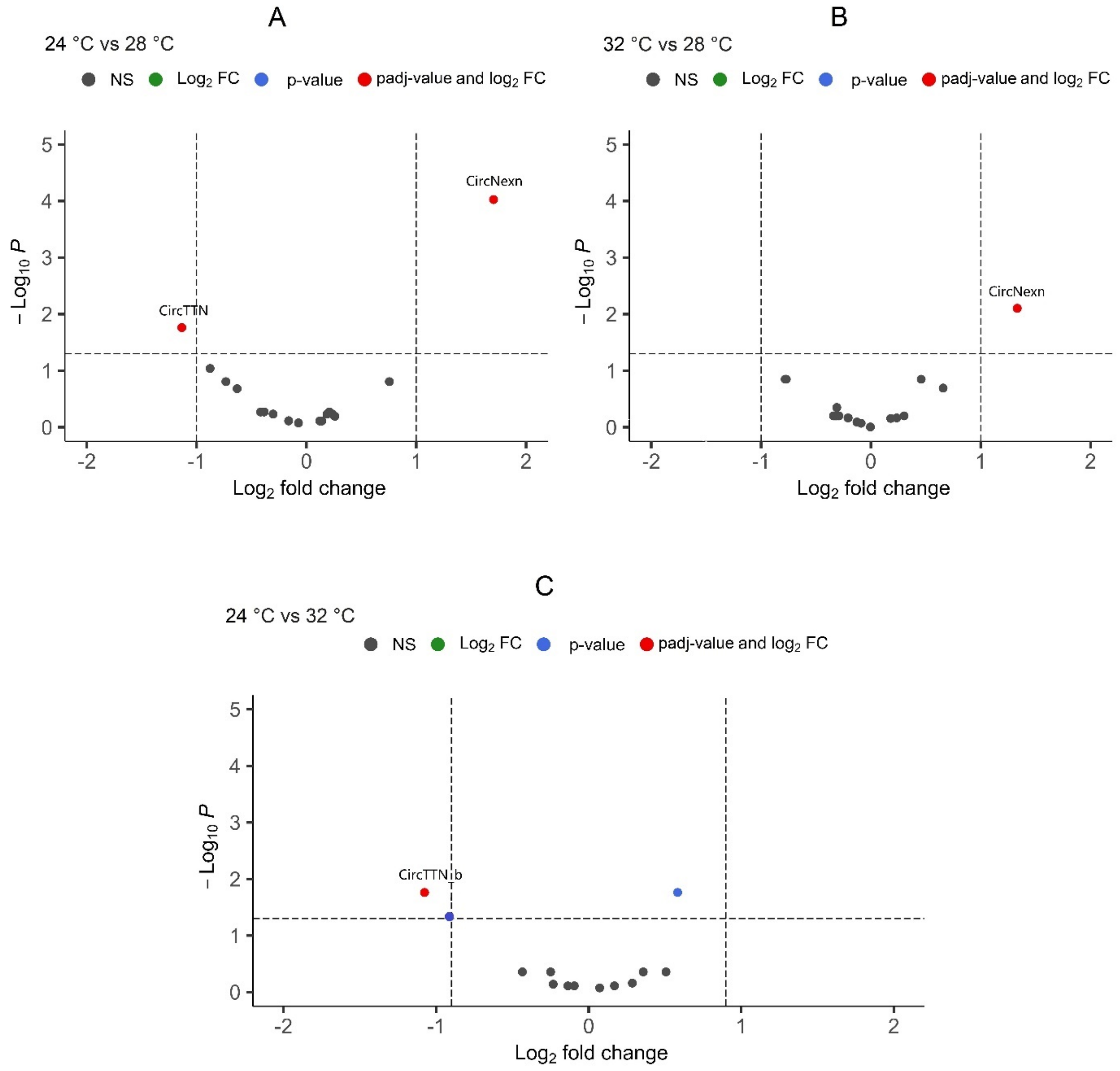

Figure S6. Volcano plots of differentially expressed circRNAs in Nile tilapia muscle. Scattered points represent genes: the x-axis is the log2fold change, and the y-axis corresponds to a Bonferroni-corrected significance value. Thresholds adjusted p-value and fold change cut-off were fixed at 0.05 and |≥| 1 (red dot). (A), (B) and (C) describe the comparisons between 24 °C vs 28 °C, 32 °C vs 28 °C and 24 °C vs 32 °C groups. Non-significant (NS) genes are marked with gray and green dots, while blue dots represent genes having a p-adjusted value below 0.5 but $|Log_2$fold change$| < 1$.

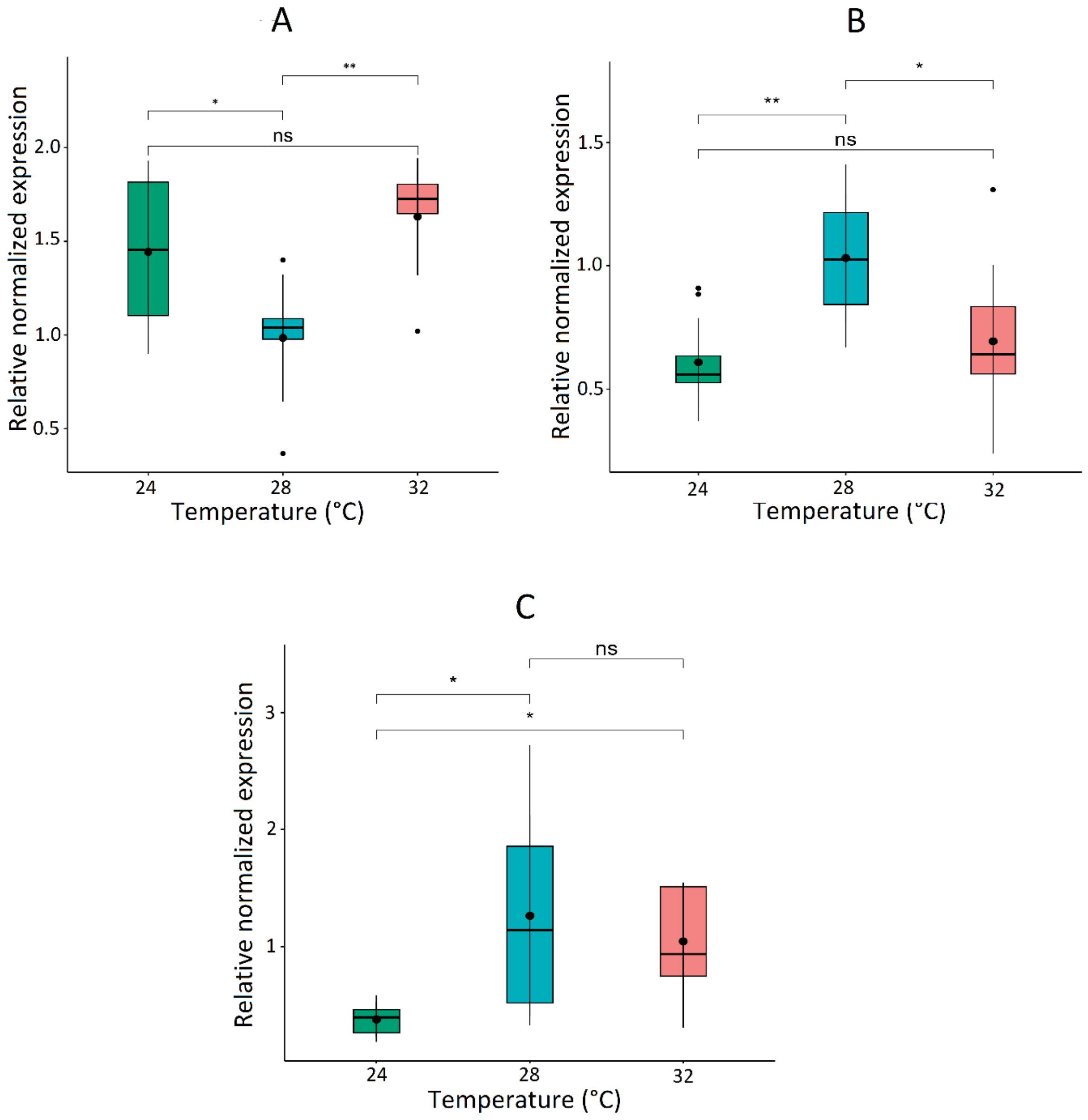

Figure S7. Relative quantification by qPCR of the differentially expressed circRNAs (A) circNexn, (B) circTTN and (C) circTTN_b. Expression levels were normalized using the ΔΔCT method, considering the geometric mean of two reference genes (*β-actin* and *elongation factor 1-alpha*). *p < 0.05;**p < 0.01
